# L-Type Ca_v_1.3 Calcium Channels Are Required for Beta-Adrenergic Triggered Automaticity in Dormant Mouse Sinoatrial Pacemaker Cells

**DOI:** 10.3390/cells11071114

**Published:** 2022-03-25

**Authors:** Julien Louradour, Olivier Bortolotti, Eleonora Torre, Isabelle Bidaud, Ned Lamb, Anne Fernandez, Jean-Yves Le Guennec, Matteo E. Mangoni, Pietro Mesirca

**Affiliations:** 1 Institut de Génomique Fonctionnelle, Université de Montpellier, CNRS, INSERM, 34090 Montpellier, France; julien.louradour@unibe.ch (J.L.); olivier.bortolotti@etu.umontpellier.fr (O.B.); eleonora.torre@igf.cnrs.fr (E.T.); isabelle.bidaud@igf.cnrs.fr (I.B.); 2 LabEx Ion Channels Science and Therapeutics (ICST), 34090 Montpellier, France; 3 PhyMedExp, Université de Montpellier, INSERM U1046, UMR CNRS, 34090 Montpellier, France; jean-yves.le-guennec@inserm.fr; 4 Mammalian Stem Cell Biology Group, Institute of Human Genetics, Université de Montpellier, CNRS, 34090 Montpellier, France; ned.lamb@igh.cnrs.fr (N.L.); anne.fernandez@igh.cnrs.fr (A.F.)

**Keywords:** cardiac pacemaker activity, adrenergic regulation, Ca_v_1.3 channels, ryanodine receptors

## Abstract

Background: Sinoatrial node cells (SANC) automaticity is generated by functional association between the activity of plasmalemmal ion channels and local diastolic intracellular Ca^2+^ release (LCR) from ryanodine receptors. Strikingly, most isolated SANC exhibit a “dormant” state, whereas only a fraction shows regular firing as observed in intact SAN. Recent studies showed that β-adrenergic stimulation can initiate spontaneous firing in dormant SANC, though this mechanism is not entirely understood. Methods: To investigate the role of L-type Ca_v_1.3 Ca^2+^ channels in the adrenergic regulation of automaticity in dormant SANC, we used a knock-in mouse strain in which the sensitivity of L-type Ca_v_1.2 α1 subunits to dihydropyridines (DHPs) was inactivated (*Ca_v_1.2^DHP−/−^*), enabling the selective pharmacological inhibition of Ca_v_1.3 by DHPs. Results: In dormant SANC, β-adrenergic stimulation with isoproterenol (ISO) induced spontaneous action potentials (AP) and Ca^2+^ transients, which were completely arrested with concomitant perfusion of the DHP nifedipine. In spontaneously firing SANC at baseline, Ca_v_1.3 inhibition completely reversed the effect of β-adrenergic stimulation on AP and the frequency of Ca^2+^ transients. Confocal calcium imaging of SANC showed that the β-adrenergic-induced synchronization of LCRs is regulated by the activity of Ca_v_1.3 channels. Conclusions: Our study shows a novel role of Ca_v_1.3 channels in initiating and maintaining automaticity in dormant SANC upon β-adrenergic stimulation.

## 1. Introduction

Automaticity is a fundamental property of the vertebrate heart. It relies on the spontaneous electrical activity originating from the sinoatrial node (SAN) [1,2]. The automaticity of SAN cells (SANC) is due to diastolic depolarization (DD), a slow depolarizing phase of the action potential (AP) cycle which drives the membrane voltage (V_m_) from the end of repolarization to the threshold of the following AP [3]. While some aspects of the pacemaker mechanism are still not fully elucidated, there is considerable agreement that SAN pacemaker activity is generated by a complex interplay between ion channels of the plasma membrane and intracellular Ca^2+^ cycling [2,4].

Several sarcolemmal ion channels contribute to SANC automaticity and their sequential activation during the DD and AP cycles forms the so-called “*membrane clock*”. Hyperpolarization-activated cyclic nucleotide-gated (HCN4) channels underlying the “funny” current (*I_f_*) [5], voltage-gated Ca^2+^ channels (VGCCs) [6,7], voltage-dependent tetrodotoxin (TTX)-sensitive Na^+^ channels [8] and transient receptor potential (TRPM and TRPC) [9,10,11] contribute to automaticity in SANC. Along with sarcolemmal ion channels activation, cyclical spontaneous ryanodine receptor-2 (RyR2)-dependent local Ca^2+^ releases (LCRs) from sarcoplasmic reticulum (SR) during DD (“*Ca*^2+^ *clock*”) also contribute to SANC automaticity [12]. The increase in intracellular calcium [Ca^2+^]_i_ by LCRs activates the Na^+^/Ca^2+^ exchanger (NCX), resulting in an enhanced inward *I_NCX_* current which accelerates the DD in late diastole. The *membrane* (*M*) *clock* and *Ca*^2+^ *clock* are both integrated in the *coupled clock* model of pacemaking, i.e., there is a self-entrainment of the two clocks to achieve sustained automaticity in SANC [4,13]. The *coupled clock* model of pacemaking has been interpreted as an ignition process: along with HCN4 activation, spontaneous LCRs and increased *I_NCX_* depolarize the cell membrane, reaching the threshold for T-type and L-type (*I_Ca,T_* and *I_Ca,L_*, respectively) Ca^2+^ currents. These diastolic Ca^2+^ influxes in turn activate more LCRs by Ca^2+^-induced Ca^2+^ release (CICR) [14]. AP upstroke is then generated when V_m_ reaches the threshold for the activation of *I_CaL_*, and repolarization is initiated by the inactivation of VGCCs and the activation of delayed outward K^+^ currents *I_Kr_* and *I_Ks_* [14]. During the AP upstroke, Ca^2+^ influx refills the SR Ca^2+^ content via the sarco/endoplasmic reticulum Ca^2+^-ATPase (SERCA), setting an appropriate Ca^2+^ load of the SR for diastolic LCRs to occur in the following DD and AP cycle [15]. In the *coupled clock* model of pacemaking, β-adrenergic activation accelerates pacemaker activity by enhancing the coupling of the two clocks in SANC, via an increase in intracellular cyclic adenosine monophosphate (cAMP) levels [4]. cAMP directly enhances *I_f_* and shifts its voltage dependence towards more depolarized potentials [16]. cAMP-mediated protein kinase A (PKA)-dependent phosphorylations of VGCCs, RyR2, and phospholamban (PLB) augment their activity which ultimately increases the depolarization rate of SANC [2,17].

Recently, β-adrenergic stimulation on isolated SANC was studied in quiescent SANC [18,19,20,21]. Kim et al. showed that the majority of isolated SANC from guinea pig hearts were dormant (i.e., with no apparent spontaneous electrical activity) and that β-adrenergic receptor activation can initiate automaticity in these cells, which start firing spontaneously [20]. In addition, dormant SANC and SANC that are spontaneously firing at baseline can reach the same frequency under β-adrenergic stimulation. This β-adrenergic effect was also observed in isolated dormant human SANC [22]. Interestingly, a recent study showed that the chronotropic response is actually different among SANC with various basal firing rates which results, ultimately, in a synchronized average rate for all SANC under β-adrenergic stimulation [23].

We have previously shown that SANC express two distinct isoforms of L-type VGCCs: Ca_v_1.3 and Ca_v_1.2 [24]. These isoforms underlie distinct L-type Ca^2+^ currents activating at different voltages. Indeed, while Ca_v_1.3-mediated *I_CaL_* (*I_Cav1.3_*) activates at negative voltages and supplies an inward current during DD, Ca_v_1.2-mediated *I_CaL_* (*I_Cav1.2_*) activates at more positive voltages in the range of the AP upstroke phase [6,7,25]. Both Ca_v_1.3 and Ca_v_1.2 are positively regulated by catecholamines [25]. Ca_v_1.3 channels also constitute an essential molecular component of the sustained inward Na^+^ current (*I_st_*) in SANC, which provides an additional mechanism to supply inward current in the DD range [26]. Furthermore, in recent works, we have shown that Ca_v_1.3 channels co-localize with RyR2 in SANC and contribute to the generation of diastolic LCRs [27,28,29]. Finally, we have shown that intact Ca_v_1.3 is necessary for the SAN impulse to be initiated in the SAN [27], making these channels a potentially important factor in the regulation of automaticity in dormant and spontaneously active SANC upon the activation of β-adrenergic receptors.

Here, we show that L-type Ca_v_1.3 channels are critical for the initiation of pacemaker activity in dormant mouse SANC by β-adrenergic activation. We show that selective pharmacologic inhibition of Ca_v_1.3 channels using mice in which the sensitivity of Ca_v_1.2 channels has been abolished (*Ca_v_1.2^DHP−/−^*) stops the automaticity of dormant SANC, reverses the positive chronotropic effect of isoproterenol (ISO) in spontaneously active SANC, and disrupts the synchronization of LCRs under the β-adrenergic stimulation of pacemaking. In addition, we suggest that Ca_v_1.3 channels are essential for the recruitment of dormant SANC under β-adrenergic stimulation to reach a synchronized rate among SANC.

## 2. Materials and Methods

All mice used were from the C57BL/6J genetic background. Wild-type (WT) mice were obtained from Charles River and Janvier labs. Ca_v_1.2^DHP−/−^ mice, obtained from J. Striessnig’s team, were generated by knock-in point mutation in Ca_v_1.2 α1 subunit (T1066Y) [30]. The study is in accordance with the Guide for the Care and Use of Laboratory Animals published by the US National Institute of Health (NIH Publication No. 85-23, revised 1996) and European directives (2010/63/EU). Experimental procedures were approved by the Ethical Committee of the University of Montpellier and the French Ministry of Agriculture (protocol no: 2017010310594939). Animals were housed in the IGF animal facility with free access to food and water and were exposed to 12 h light/dark reverse cycles (light, 8 p.m. to 8 a.m.) in a thermostatically controlled room (21–22 °C).

### 2.1. Single Cell Isolation

SAN pacemaker cells were isolated from both male and female WT and Ca_v_1.2^DHP−/−^ mice hearts as previously described [27,31]. Briefly, the heart was excised and immersed in a pre-warmed (36 °C) Tyrode solution containing 140 mM NaCl, 5.4 mM KCl, 1 mM MgCl_2_, 1.8 mM CaCl_2_, 5.5 mM D-glucose, and 5 mM Hepes (adjusted to PH 7.4 with NaOH). SAN region was identified using the superior and inferior *vena cava*, the crista terminalis, and the interatrial septum as landmarks. SAN tissue was excised and transferred into a low-Ca^2+^ solution containing 140 mM NaCl, 5.4 mM KCl, 0.5 mM MgCl_2_, 0.2 mM CaCl_2_, 1.2 mM KH_2_PO_4_, 50 mM taurine, 5.5 mM D-glucose, 1 mg/mL BSA, and 5 mM Hepes–NaOH (adjusted to pH 6.9 with NaOH) for 4 min. Then, enzymatic digestion was carried out for 15–20 min at 36 °C in the low-Ca^2+^ solution containing purified collagenase 1 and II (0.15 mg/mL Liberase medium Thermolysin, Roche, Mannheim, Germany) and elastase (0.5 mg/mL, Worthington, Lakewood, NJ, USA). Digestion was stopped by washing the SAN in a “Kraftbrühe” (KB) medium containing 100 mM K-glutamate, 10 mM K-aspartate, 25 mM KCl, 10 mM KH_2_PO_4_, 2 mM MgSO_4_, 20 mM taurine, 5 mM creatine, 0.5 mM EGTA, 20 mM D-glucose, 5 mM Hepes, and 1 mg/mL BSA (adjusted to PH 7.2 with KOH). Single SAN cells were then dissociated from the SAN tissue by manual agitation using a flame-forged Pasteur’s pipette. To recover the automaticity of the SAN cells, Ca^2+^ was gradually reintroduced in the cell’s storage solution to a final concentration of 1.8 mM [31]. SAN cells were then left to rest for 1 h before recording.

### 2.2. Patch-Clamp Recordings of SAN Cells

SAN cells were harvested in custom-made chambers with glass bottoms for cell attachment and superfused with Tyrode solution warmed at 36 °C before recording. All electrophysiological signals, APs and ion currents, were measured by perforated patch-clamp technique and recorded using a Multiclamp 700 A patch clamp amplifier connected to Digidata 1550B interface (Molecular Devices). Electrodes had a resistance of 3–4 MΩ when filled with a solution containing (in mM): 80 K-aspartate, 50 KCl, 1 MgCl_2_, 2 CaCl_2_, 5 EGTA, 5 HEPES, and 3 ATP-Na (adjusted to pH 7.2 with KOH). Perforated patch-clamp was performed by adding 30 µM β-escin to the intracellular solution. Seal resistances were in the range of 2–5 GΩ. Perfusion of pre-warmed (36 °C) experimental solutions was achieved by using a multi-MPRE8 heating pen (Cell Micro Controls). AP parameters were measured as previously described (see Supplementary Table S1 inset in ref. [27]). For ion current measurements, dormancy or firing of SAN cells at baseline were recorded in current-clamp for at least 30 s before switching to voltage-clamp.

#### 2.2.1. I_CaL_ Measurement

To record I_CaL_, 30 µM tetrodotoxin (TTX) was added to Tyrode perfusion. From a holding potential of −55 mV, depolarizing steps of 80 ms were undertaken from −55 to +60 mV. Peak density was taken and plotted against corresponding voltage (I–V curve) for each cell. Nifedipine (3 µM) was added to Tyrode + TTX perfusion to inhibit L-type Ca_v_1.3 Ca^2+^ channels in isolated *Ca_v_1.2^DHP−/−^* SANC. Nifedipine-sensitive current was obtained by withdrawing the current recorded in nifedipine condition to the one recorded in Tyrode + TTX condition.

#### 2.2.2. I_f_ Measurement

Hyperpolarizing steps from −135 to −35 mV in +10 mV increments were undertaken from a holding potential of −35 mV. Each hyperpolarizing step lasted 1700 ms and IV curves were plotted for each cell with current density taken at steady state.

### 2.3. 2D Calcium Imaging of Single SAN Cells

Calcium dynamics in isolated SAN cells were measured with confocal microscopy (Zeiss LSM 780) as previously described [29]. Briefly, SAN cells were seeded in a glass Fluorodish (FD3510-100, WPI), coated overnight with laminin (1 mg/mL, Sigma-Aldrich, Saint-Louis, MO, USA), for 1 h before recordings. Cells were loaded for 20 min with a Tyrode solution containing the Ca^2+^ indicator CAL-520 (1 ng/µL). After washing CAL-520 two times, cells were recorded in Tyrode bath solution. Images were acquired by scanning SAN cells with an argon laser in line-scan configuration. Fluorescence was excited at 488 nm and emissions were collected at >505 nm. A 63× oil immersion objective was used to record [Ca^2+^]_i_ in isolated SAN cells. Perfusion of pre-warmed (36 °C) experimental solutions was achieved by using a multi-MPRE8 heating pen (Cell Micro Controls). SR Ca^2+^ load was measured by acute addition of 10 mM caffeine to bath solution. Background noise was suppressed and the time-course of Ca^2+^ fluorescence was analyzed with Clampfit (ver. 11.1). Ca^2+^ transients were analyzed using ImageJ software but LCRs were analyzed using a homemade software written on R (olivierbortolotti/lcr-analysis-san (github.com), accessed on 22 November 2021), inspired by IOCBIO sparks detection and analysis software [32]. Since cells have not been permeabilized, the software measured LCR parameters after suppressing the AP-induced Ca^2+^ transient to allow an accurate comparison between dormant and firing SANC.

### 2.4. Intact SAN Immunostaining

Intact SAN–atria preparations were obtained as previously described [27]. Briefly, entire SAN–atria preparation was placed in pre-warmed (36 °C) Tyrode solution and pinned to the bottom of an optical chamber (Fluorodish, FD35PDL-100; WPI) coated with ~2 mm of clear Sylgard (Sylgard 184 Silicone elastomer kit; Dow Corning, Midland, TX, USA). To maintain the SAN in a flat plane, right and left atria were also pinned using entomology needles. Superior and inferior vena cava were then cut open to expose the SAN. SAN preparation was fixed for 20 min at 4 °C with 4% paraformaldehyde in phosphate buffer (PBS), washed twice with PBS, and permeabilized with cold (−20 °C) methanol for 5 min. Sample was again washed twice and then incubated for 1 h with blocking solution (10% goat serum, 1% BSA and 0.05% NaN_3_ in PBS). SAN was incubated with anti-Ca_v_1.3 (rabbit, 1:200, obtained from [33] and anti-HCN4 (guinea pig, 1:200, Alomone labs, Cat. AGP-004) primary antibodies at 4 °C overnight. Sample was washed and incubated for 1 h at 37 °C with Alexa 488 goat anti-rabbit (1:200, Thermo Fischer Scientific, Waltham, MA, USA, Cat. A11034) and Alexa 594 goat anti-guinea pig (1:200, Abcam, Cat. ab150188) secondary antibodies. SAN preparation was washed twice and mounted with Prolong diamond antifade reagent (Thermo Fischer Scientific, Waltham, MA, USA, Cat. P36961). Images were captured with a Leica DM1 microscope.

### 2.5. Ca_v_1.3 and HCN4 Labeling in Isolated SANC

SANC were isolated as described above and plated on a multi-well glass slide (Lab-Tek Chamber slide^TM^, Thermo Fischer Scientific), coated with 0.1 M NaHCO_3_, 15 mM NaOH and 50 µg·mL^−1^ Cell tak (Corning^TM^ Cell Tak, Thermo Fischer Scientific) for at least 20 min. Cells were fixed for 15 min at room temperature with 4% paraformaldehyde in PBS, washed twice with PBS, and then permeabilized for 15 min with 0.1% Triton in PBS. Samples were again washed with PBS and incubated for 1 h with the blocking solution (see above). SANC were incubated at 4 °C overnight with anti-Ca_v_1.3 and anti-HCN4 (see above) primary antibodies and then incubated with Alexa 488 goat anti-rabbit and Alexa 594 goat anti-guinea pig (see above) for 1 h at 37 °C. Cells were finally washed twice and mounted with Prolong diamond antifade reagent (Thermo Fischer Scientific, Cat. P36961). Images were captured with an Axio Imager Z1 Apotome microscope (Carl Zeiss, Iéna, Germany).

### 2.6. Dual Immunostaining of Phospholamban in Isolated SANC

To investigate the potential role of Ca_v_1.3 in phospholamban (PLB) phosphorylation on serine 16 or threonine 17, isolated SANC needed to be in a physiological solution. Therefore, immunostaining was completed after Ca^2+^ reintroduction (see single cell isolation protocol above). SANC were plated on a multi-well glass slide (see above) and incubated for 5 min in Tyrode solution. Cells were separated in three groups and incubated with: condition 1: Tyrode for 6 min; condition 2: 100 nM isoproterenol (ISO) for 6 min; or condition 3: 100 nM ISO for 3 min and then ISO + 3 µM nifedipine (Nife) for 3 min. Immunostaining was then performed as described above with anti-PLB total (mouse, 1:200, Badrilla, Cat. No. A010-14) and anti-phosphorylated PLB at serine 16 (rabbit, 1:200, Badrilla, Cat. No. A010-12AP) or anti-phosphorylated PLB at threonine 17 (rabbit, 1:200, Badrilla, Cat. No. A010-13) primary antibodies and Alexa 594 goat anti-mouse (1:200, Jackson laboratories, Cat. 115-585-003) and Alexa 488 goat anti-rabbit (1:200, Thermo Fischer Scientific, Cat. A11034) secondary antibodies.

### 2.7. Statistical Analysis

Statistical analysis was performed using Prism 9.1.2 (GraphPad Software). Data are expressed as mean ± the standard error of the mean (SEM). *p* ≤ 0.05 was considered statistically significant. Statistical tests used in each experiment are specified throughout the figure legends.

## 3. Results

### 3.1. Inhibition of L-Type Ca^2+^ Channels Reversed Adrenergic-Induced Acceleration of AP Frequency in Isolated SANC from WT Mice

**Figure 1:**
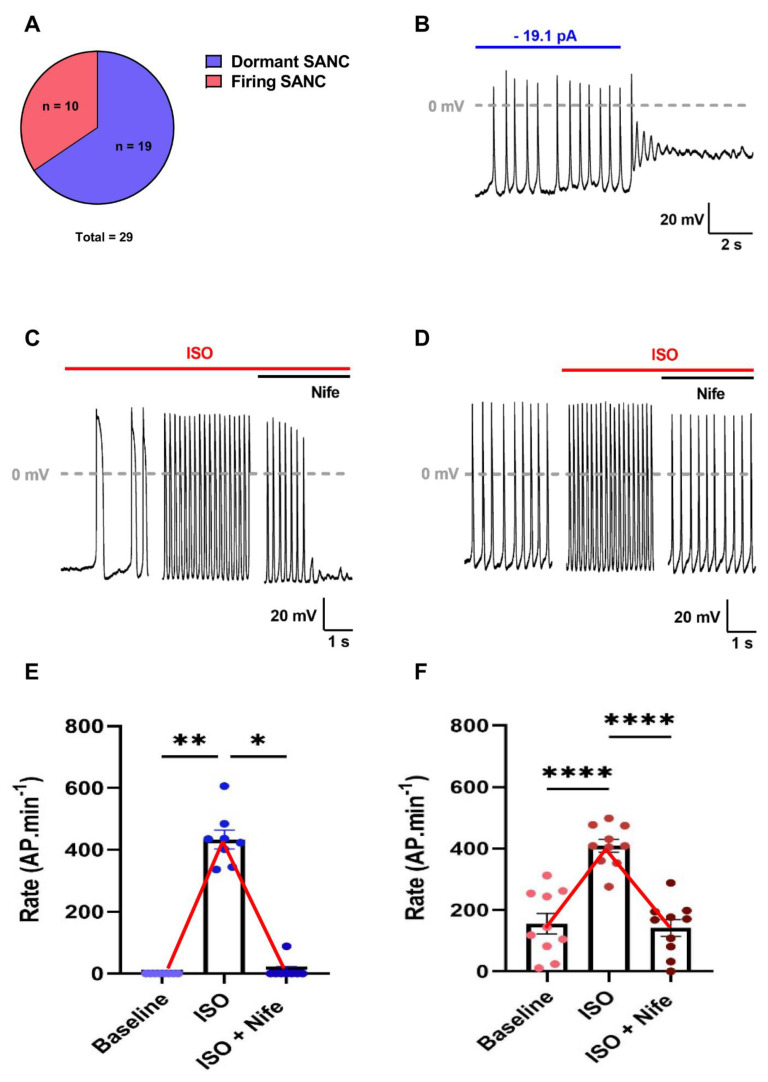
L-type Ca^2+^ channels inhibition completely reversed the ISO-induced acceleration of depolarization rate in both dormant and spontaneously firing SANC isolated from WT mice. (**A**) Proportion of dormant and spontaneously firing SANC at baseline. (**B**) Current-clamp recording sample of dormant SANC initiating firing when injected with hyperpolarizing current. Average depolarization rates and illustrative examples of dormant ((**C**–**E**), n = 8) and firing ((**D**–**F**), n = 10) SANC at baseline and after perfusion of isoprenaline (ISO 100 nM) and subsequent perfusion of nifedipine (3 µM). * *p* < 0.05, ** *p* < 0.01, **** *p* < 0.0001 by parametric one-way ANOVA or non-parametric Friedman test.

We studied spontaneous AP firing in n = 29 SANC isolated from six WT mouse hearts. Of the 29 cells, two-thirds exhibited a dormancy behavior at baseline (dormant SANC) and a third were spontaneously firing (firing SANC) (Figure 1A). Dormant cells had no apparent spontaneous electrical activity. Their resting potential was either depolarized around −30 mV or hyperpolarized around −70 mV. However, they showed spontaneous AP firing when injected with artificial current to reach membrane potentials falling within the range of the diastolic depolarization of firing SANC (Figure 1B, Supplementary Figure S1B). Firing SANC presented a variable degree of rhythmicity, evidenced by the dispersion of the coefficient of variation (standard deviation/mean, CoV) of the firing inter-beat time (Supplementary Figure S1C).

Of the 19 dormant SANC, 8 initiated firing under β-adrenergic stimulation (100 nM Isoprenaline, ISO), reaching beating frequencies of 433 ± 30 bpm, while 11 remained dormant (Figure 1C,E, Supplementary Figure S1A). The resting potential of dormant SANC that did not initiate firing was significantly depolarized compared with the ones that started firing (Supplementary Figure S1D). In firing SANC, ISO significantly augmented the frequency by increasing the linear and exponential slope of the DD and decreasing AP duration without affecting any other AP parameters (Supplementary Table S1). β-adrenergic stimulation also brought all SANC to the same level of rhythmicity (CoV: 0.04 ± 0.01 for both dormant and firing SANC). In addition, dormant SANC reached beating frequencies similar to firing SANC under ISO (433 ± 30 and 409 ± 21 bpm, respectively) (Supplementary Table S1).

To investigate the role of L-type Ca^2+^ channels in adrenergic-induced firing in dormant SANC, we additionally superfused cells with 3 µM nifedipine (Nife), a concentration ensuring the full blockade of L-type Ca^2+^ currents [26]. We observed that L-type Ca^2+^ channel inhibition completely stopped AP firing under β-adrenergic stimulation (Figure 1C,E). In contrast, Nife did not stop AP firing in firing SANC but significantly decreased the firing frequency, which returned to values similar to basal before stimulation (Tyrode: 156 ± 33 bpm; ISO + Nife: 142 ± 27 bpm), despite continuous β-adrenergic stimulation (Figure 1D,F, Supplementary Figure S1A).

In conclusion, the inhibition of L-type Ca^2+^ channels completely reversed the positive chronotropic effect of β-adrenergic stimulation in both dormant and firing SANC. These data indicate that L-type Ca^2+^ channels are required for the initiation of automaticity in dormant SANC and play a major role in accelerating the beating frequency in firing SANC upon β-adrenergic activation.

### 3.2. Selective Inhibition of L-Type Ca_v_1.3 Ca^2+^ Channels Reversed Adrenergic-Induced Acceleration of AP Frequency in Isolated SANC of Ca_v_1.2^DHP−/−^ Mice

**Figure 2:**
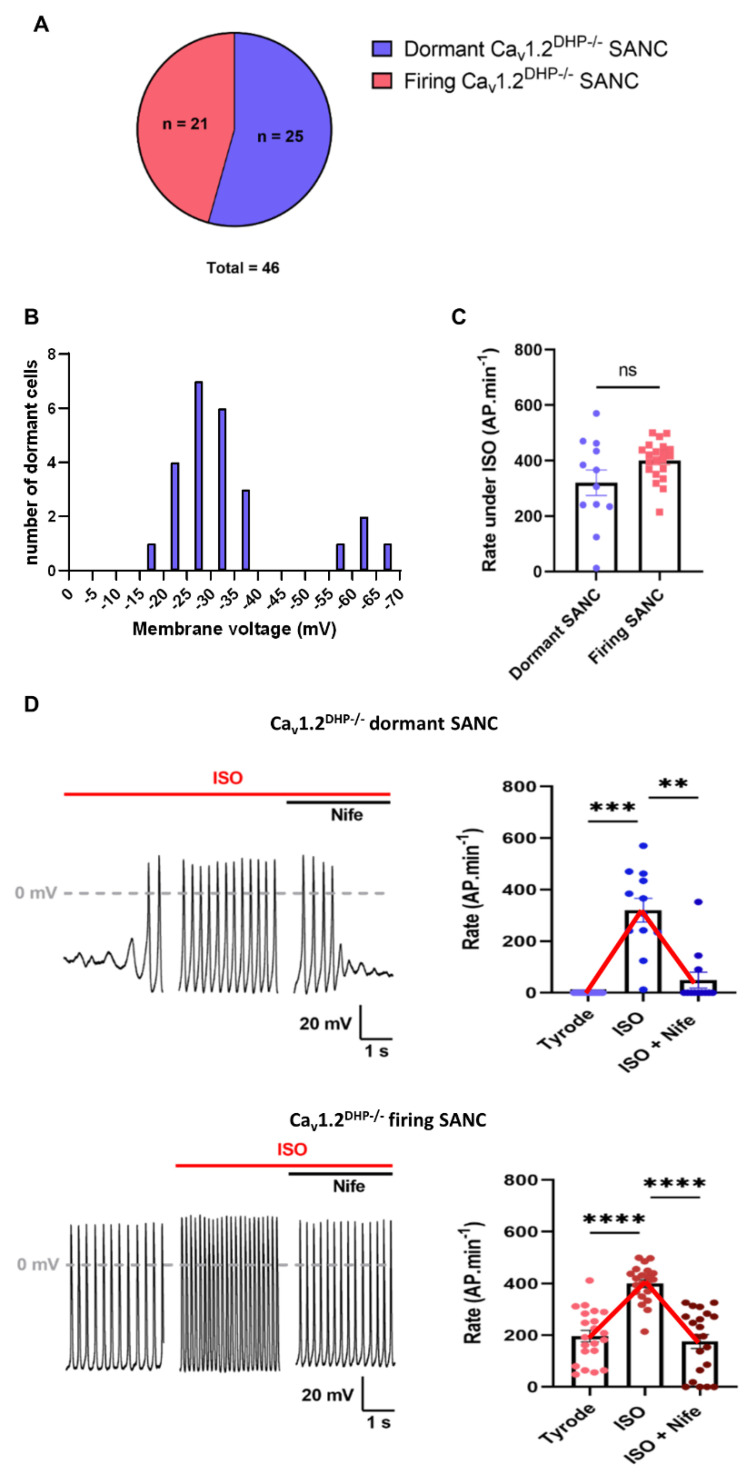
Ca_v_1.3 Ca^2+^ channels are required for ISO-induced acceleration of depolarization rate in dormant and spontaneously firing SANC isolated from *Ca_v_1.2^DHP−/−^* mice. (**A**) Proportion of dormant and spontaneously firing *Ca_v_1.2^DHP−/−^* SANC at baseline. (**B**) Distribution of membrane potential of dormant *Ca_v_1.2^DHP−/−^* at baseline. (**C**) Firing rate comparison between dormant (n = 12) and firing (n = 21) *Ca_v_1.2^DHP−/−^* SANC under adrenergic stimulation (ISO). (**D**) Average firing rates and illustrative examples of dormant (n = 12) and firing (n = 21) *Ca_v_1.2^DHP−/−^* SANC at baseline and after perfusion ISO (100 nM) and subsequent perfusion of ISO and Nife (3 µM). ** *p* < 0.01, *** *p* < 0.001, **** *p* < 0.0001 by non-parametric Friedman test.

Previous work has shown that Ca_v_1.3 channels contribute to the generation of DD [25,27,29]. We thus attempted to determine how Ca_v_1.3 channels were involved in the adrenergic-induced initiation of automaticity in dormant SANC. We studied spontaneous firing in SANC isolated from 16 *Ca_v_1.2^DHP−/−^* mouse hearts. In *Ca_v_1.2^DHP−/−^* mice, a T1066Y knock-in point mutation in the Ca_v_1.2 α1 subunit inactivates its sensitivity to dihydropyridines, allowing the selective pharmacological inhibition of Ca_v_1.3 channels by Nife [30]. Of the 46 cells studied, 21 were spontaneously firing and 25 were dormant at baseline, showing roughly the same proportion as WT SANC (Figure 1A and Figure 2A).

At baseline, *Ca_v_1.2^DHP−/−^* firing SANC showed no significant difference in AP parameters from WT firing SANC, except for the exponential slope of diastolic depolarization, which was higher, and the AP threshold, which was more negative, in *Ca_v_1.2^DHP−/−^* cells (Supplementary Table S2). As observed with WT dormant SANC, the resting membrane potential of dormant *Ca_v_1.2^DHP−/−^* SANC laid positive to −35 mV, outside of the DD range (−60 to −40 mV) (Figure 2B). Of the 25 *Ca_v_1.2^DHP−/−^* dormant SANC, 12 started firing under β-adrenergic stimulation, among which nine completely stopped firing after subsequent perfusion of 3 µM Nife (Figure 2D). Similar to dormant WT SANC, the resting potentials of dormant *Ca_v_1.2^DHP−/−^* SANC that did not fire under ISO perfusion were significantly depolarized (Supplementary Figure S2H). The resting potential of dormant SANC that stopped firing under concomitant perfusion of ISO and Nife was significantly depolarized compared with the MDP under ISO conditions alone, but did not return to the basal depolarized membrane potential (Supplementary Figure S2I). In firing *Ca_v_1.2^DHP−/−^* SANC, ISO perfusion significantly increased the spontaneous frequency, and the linear and exponential slope of the DD, and decreased AP duration. Subsequent perfusion of Nife reversed the effects of the adrenergic stimulation on the above parameters, except for AP duration, and converted rhythmic firing to dysrhythmic (CoV, ISO: 0.06 ± 0.01; ISO + Nife: 0.47 ± 0.15) (Figure 2D, Table 1).

Spontaneous firing under β-adrenergic stimulation in dormant and firing *Ca_v_1.2^DHP−/−^* SANC was different. Indeed, while frequency, AP duration and the linear slope of the DD showed no statistical difference, dormant SANC APs under ISO exhibited a depolarized MDP and AP threshold, with a slower exponential slope of DD and AP upstroke and smaller amplitude compared with firing SANC (Supplementary Figure S2A–G). Taken together, these data show that the selective inhibition of Ca_v_1.3 channels can completely reverse the effects of β-adrenergic stimulation in both dormant and firing *Ca_v_1.2^DHP−/−^* SANC.

In addition, we did not find significant differences in the characteristics of AP firing between WT and *Ca_v_1.2^DHP−/−^* SANC under all conditions tested, indicating that the observed effects in β-adrenergic stimulation are attributable to Ca_v_1.3 channels (Supplementary Table S2).

### 3.3. Selective Inhibition of L-Type Ca_v_1.3 Ca^2+^ Channels Disrupts Ca^2+^ Release in Isolated Ca_v_1.2^DHP−/−^ SANC

**Figure 3:**
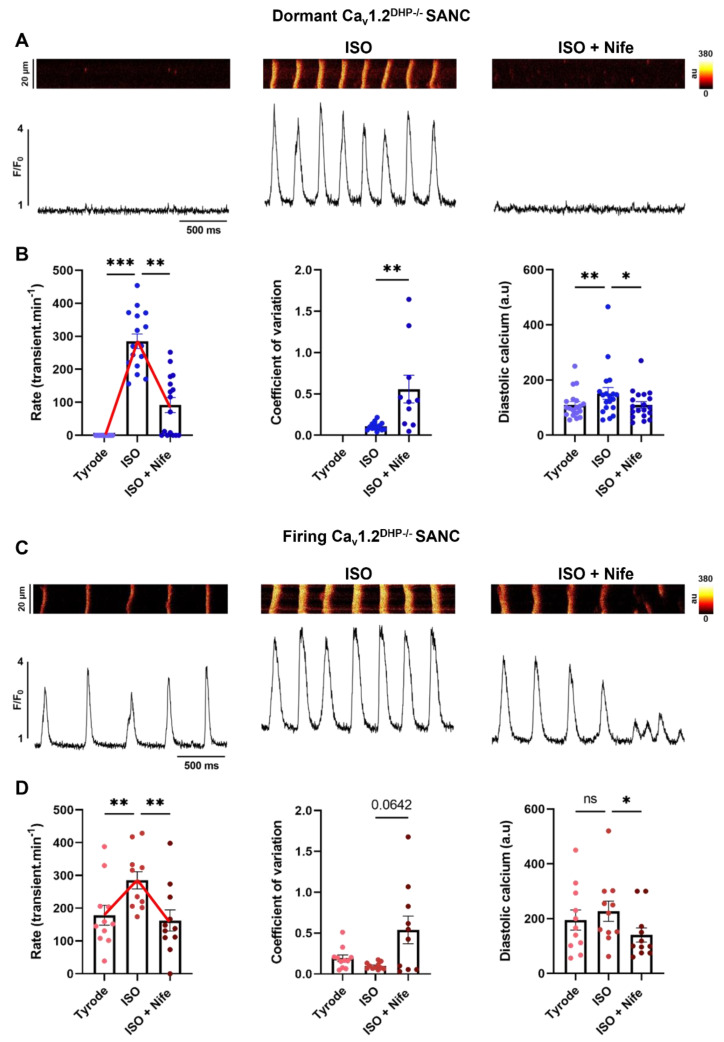
Inhibition of Ca_v_1.3 Ca^2+^ channels decreased spontaneous Ca^2+^ transient rate under β-adrenergic stimulation in isolated *Ca_v_1.2^DHP−/−^* SANC. Line-scan and corresponding time-course of Ca^2+^ fluorescence in dormant (**A**) and spontaneously firing (**C**) *Ca_v_1.2^DHP−/−^* SANC in Tyrode, ISO (100 nM) and ISO + Nife (3 µM) conditions. Average Ca^2+^ transient rate, mean coefficient of variation and mean diastolic calcium in dormant ((**B**), blue, n = 16) and firing ((**D**), red, n = 11) *Ca_v_1.2^DHP−/−^* SANC. * *p* < 0.05, ** *p* < 0.01, *** *p* < 0.001 by parametric one-way ANOVA or non-parametric Friedman test.

Since intracellular calcium dynamics contribute to physiological pacemaking, we performed 2D line-scan calcium imaging on 30 SANC isolated from 13 *Ca_v_1.2^DHP−/−^* hearts. Of the 30 cells, two-thirds were dormant (i.e., cells exhibiting only LCRs with no spontaneous Ca^2+^ transients) and one-third were spontaneously firing at baseline (i.e., cells with LCRs and spontaneous Ca^2+^ transients) (Figure 3A,C). A few cells were difficult to classify as dormant or firing, since they presented with a very low frequency of spontaneous activity. Hence, we classified cells showing spontaneous frequency below 5 transients/min as dormant. Of the 19 dormant SANC, only three (16%) failed to initiate spontaneous firing under β-adrenergic stimulation, a reduced percentage compared with current clamp experiments (*p* = 0.025, Chi square test) (Figure 1 and Figure 2). Notably, the percentage of dormant SANC that arrested firing under subsequent perfusion of Nife was also lower (5 out 16 compared with 9 out of 12, *p* = 0.02 Chi square test) (Figure 2 and Figure 3B). In firing SANC, β-adrenergic stimulation significantly increased the frequency of Ca^2+^ transients, while the concomitant perfusion of ISO and Nife induced a return to basal levels (Tyrode: 179 ± 31; ISO + Nife: 162 ± 32 bpm) (Figure 3C,D). The inhibition of Ca_v_1.3 also converted β-adrenergic rhythmic firing to dysrhythmic in both dormant and firing SANC (Figure 3B,D). Consistent with what we observed in current clamp experiments, the frequency of Ca^2+^ transients under β-adrenergic stimulation was similar between dormant and firing SANC (respectively 285 ± 22 and 285 ± 26 bpm) (Figure 3B,D).

To determine the effects of Ca_v_1.3 inhibition on Ca^2+^ dynamics, we analyzed the properties of Ca^2+^ transients and LCR parameters in *Ca_v_1.2^DHP−/−^* SANC. ISO significantly increased diastolic intracellular calcium ([Ca^2+^]_i_) levels in dormant but not in firing SANC. Ca_v_1.3 inhibition significantly decreased the diastolic level of [Ca^2+^]_i_ in dormant and firing SANC (Figure 3B,D). While the number of LCRs increased under β-adrenergic stimulation and decreased after subsequent perfusion of Nife in dormant SANC, there was no significant difference in firing SANC (Table 2). β-adrenergic stimulation also increased the LCR size and Ca_v_1.3 inhibition decreased it in both dormant and firing SANC (Table 2). In contrast, there was no difference in LCR amplitude and duration in neither dormant nor firing SANC (Table 2).

Interestingly, three dormant SANC did not initiate firing under β-adrenergic stimulation. In the sac line, some LCRs can fall very close to the Ca^2+^ transient and even merge with it, rendering the proper detection and counting of these LCRs difficult. However, since these dormant SANC did not exhibit spontaneous Ca^2+^ transients, direct effects of β-adrenergic stimulation and subsequent Ca_v_1.3 inhibition on single LCRs could be observed. In these cells, ISO increased the number of LCRs while subsequent Ca_v_1.3 inhibition decreased it. Ca_v_1.3 inhibition also decreased LCR size while no trend could be identified for LCR duration and amplitude (Supplementary Figure S3).

Taken together, these results indicate that the disruption of diastolic [Ca^2+^]_i_, and the generation and size of LCRs by Ca_v_1.3 inhibition contribute to reverse the effects of the β-adrenergic stimulation of spontaneous activity in SANC.

### 3.4. β-Adrenergic Activation of SANC Automaticity Synchronizes LCRs in Late Diastole and Is Dependent of L-Type Ca_v_1.3 Ca^2+^ Channels

**Figure 4:**
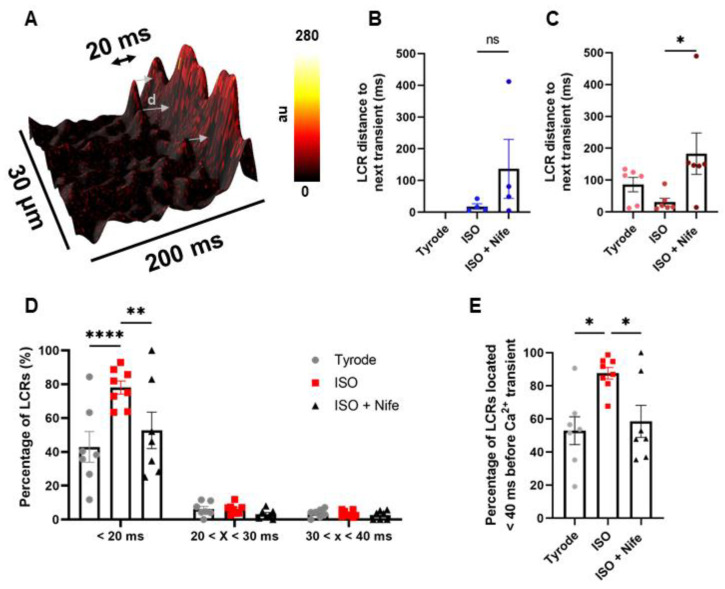
L-type Ca_v_1.3 Ca^2+^ channels control LCR synchronization upon β-adrenergic stimulation in late diastole. (**A**) Three-dimensional reconstruction of diastolic [Ca^2+^]_i_ increase with represented distance (d) between LCR and following Ca^2+^ transient. Average distance d (in ms) in dormant (**B**), n = 4) and firing (**C**), n = 6) SANC. (**D**) Proportion of LCRs located < 20 ms, between 20 and 30 ms, and between 30 and 40 ms before Ca^2+^ transient in firing (n = 7) SANC in Tyrode, ISO 100 nM and ISO + Nife 3 µM conditions. (**E**) Proportion of LCRs located < 40 ms before Ca^2+^ transient. ns: non-significant, * *p* < 0.05 by one-way ANOVA, ** *p* < 0.01, **** *p* < 0.0001 by two-way ANOVA.

We previously showed that the genetic ablation of Ca_v_1.3 channels disrupts the generation and dynamics of diastolic LCRs in mouse SANC [27,29]. However, the consequences of the selective inhibition of Ca_v_1.3 on diastolic LCRs have not been studied. LCR synchronization in space and time in late DD is an important concept of the *coupled clock* model of peacemaking [4]. Therefore, the Ca_v_1.3-mediated synchronization of LCRs may contribute to explain ISO-induced spontaneous firing in dormant SANC and the positive chronotropic effect of adrenergic activation in firing SANC. We thus investigated the synchronization of diastolic LCRs in our line-scan experiments on isolated mouse SANC before and after the inhibition of Ca_v_1.3 channels. To this aim, we measured the distance in time between every single LCR event and the following Ca^2+^ transient (Figure 4A). Averaged data for each investigated cell are shown in Figure 4B,C. In dormant *Ca_v_1.2^DHP−/−^* SANC that initiated firing under β-adrenergic activation and did not stop after Nife perfusion, the average LCR–transient distance tends to increase when Ca_v_1.3 channels are inhibited compared with ISO alone (ISO: 18.3 ± 8.4; ISO + Nife: 136.8 ± 93 ms) (Figure 4B). In firing *Ca_v_1.2^DHP−/−^* SANC, β-adrenergic activation significantly decreased this distance while subsequent Ca_v_1.3 inhibition significantly increased it (Tyrode: 86.2 ± 22.5; ISO: 30.9 ± 12.1; ISO + Nife: 183.2 ± 65.1 ms) (Figure 4C).

To confirm these results, we measured the percentage of LCRs located in the last fraction of DD (i.e., <20, 30, and 40 ms before Ca^2+^ transient) in firing *Ca_v_1.2^DHP−/−^* SANC. Under basal conditions, 40% of LCRs were found in a time window shorter than 20 ms before spontaneous Ca^2+^ transient. This fraction increased to 80% under β-adrenergic stimulation and decreased to 50% with subsequent Ca_v_1.3 inhibition (Figure 4D). Interestingly, while 90% of LCRs fell within 40 ms before Ca^2+^ transient under β-adrenergic stimulation, this proportion was ≈50% under basal conditions and the concomitant perfusion of ISO and Nife, indicating that half of LCR events under these conditions were temporally unrelated to the generation of Ca^2+^ transients (Figure 4E).

To minimize the bleaching of the Ca^2+^ indicator, the acquisition of line scan images of SANC was performed at steady state under control conditions, ISO and concomitant perfusion of ISO and Nife. However, we observed an early effect of Nife perfusion in n = 2 *Ca_v_1.2^DHP−/−^* SANC just before they stopped spontaneous activity. We determined LCRs synchronization in the short window between the moment when Ca^2+^ transient frequency started decreasing and the moment when they totally disappeared. For the two cells, Ca_v_1.3 inhibition increased the average distance of LCRs to the following Ca^2+^ transient and decreased the proportion of events at a time <20 ms before the Ca^2+^ transient (Supplementary Figure S4). Therefore, LCRs became desynchronized right before the cells stopped spontaneous activity.

Taken together, these results indicate that Ca_v_1.3 Ca^2+^ channels regulate the synchronization of LCRs in late DD under β-adrenergic stimulation.

### 3.5. L-Type Ca_v_1.3 Ca^2+^ Channels Are Not Necessary for PKA-Dependent Phosphorylation of PLB under β-Adrenergic Stimulation

**Figure 5:**
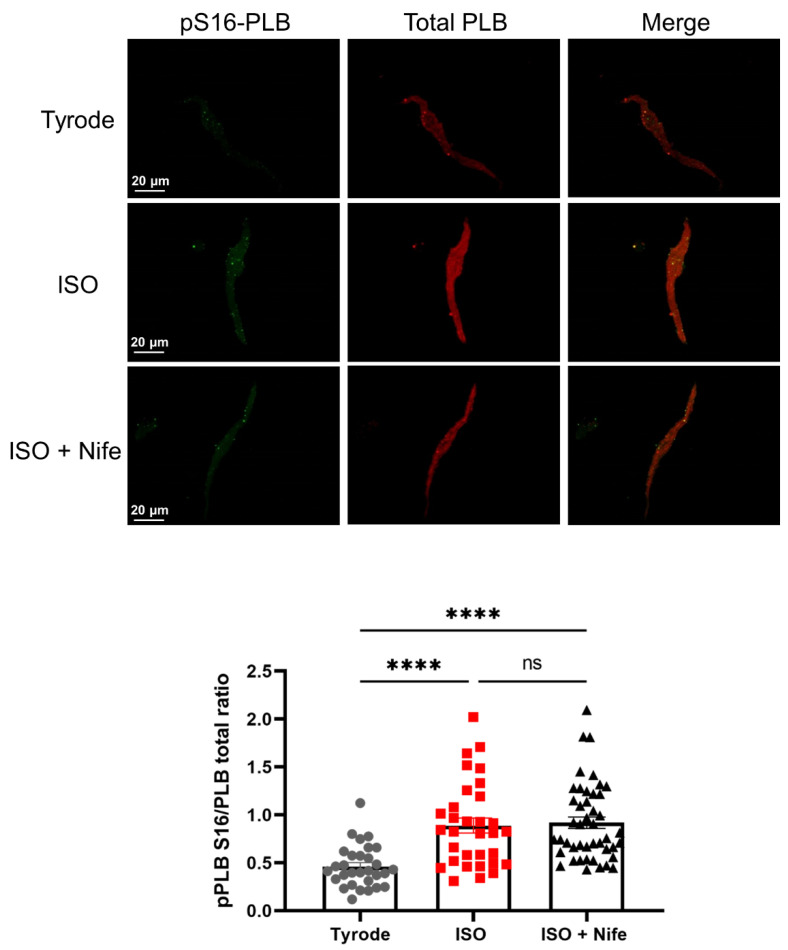
LCR regulation by Ca_v_1.3 Ca^2+^ channels is not due to a decrease in phosphorylated PLB levels. Double immunostaining of phosphorylated PLB (pPLB) at serine 16 (green), total PLB (red) and merged images in SANC incubated with Tyrode (n = 30), ISO 100 nM (n = 32) or ISO + Nife 3 µM (n = 45) with mean pPLB/PLB ratio (bottom). ns: non-significant, **** *p* < 0.0001 by one-way ANOVA test.

Previous work has shown that the activity of sarcoplasmic reticulum Ca^2+^ ATPase (SERCA) is involved in the determination of LCR phase and the responsiveness of pacemaker activity to adrenergic activation [15]. SERCA function is regulated by phospholamban (PLB), a protein that can be phosphorylated in serine 16 (pS16-PLB) by cAMP-dependent protein kinase A (PKA) and in threonine 17 (pThr17-PLB) by Ca^2+^-calmodulin kinase II (CaMKII). It has been proposed that the phosphorylation of PLB is an important factor in the ISO-induced acceleration of pacemaking (see Lakatta et al. for review) [4]. Since selective Ca_v_1.3 inhibition by Nife arrests the pacemaking of dormant SANC initiated by β-adrenergic activation and reverses its positive chronotropic effect in firing SANC, we investigated if Nife would also affect the phosphorylation state of PLB when concomitantly perfused with ISO (Figure 5).

We quantified the pS16-PLB/total PLB and pThr17-PLB/total PLB ratio in isolated SANC from 3 *Ca_v_1.2^DHP−/−^* mice by immunostaining each in basal conditions or after incubation with 100 nM ISO alone or concomitant incubation with ISO and 3 µM Nife (ISO + Nife). In response to β-adrenergic stimulation, the pS16-PLB/total PLB ratio increased twofold. Subsequent incubation with Nife did not affect the phosphorylation level of PLB, which remained two times higher than in basal conditions (Figure 5). pThr17-PLB/total PLB ratio was increased by 45% under the ISO condition. pThr17-PLB/total PLB ratio slightly decreased upon concomitant incubation with ISO and Nife. However, we did not observe a reversal to the basal ratio level and the pThr17-PLB/total PLB ratio remained 20% higher than in the control (Supplementary Figure S5). Therefore, while the effects of Ca_v_1.3 inhibition under β-adrenergic stimulation on LCRs may also involve a reduction in CaMKII-dependent PLB phosphorylation, PKA-dependent phosphorylation was not affected by Ca^2+^ channel blockade.

### 3.6. Diminished Diastolic Ionic Currents in Dormant SANC

**Figure 6:**
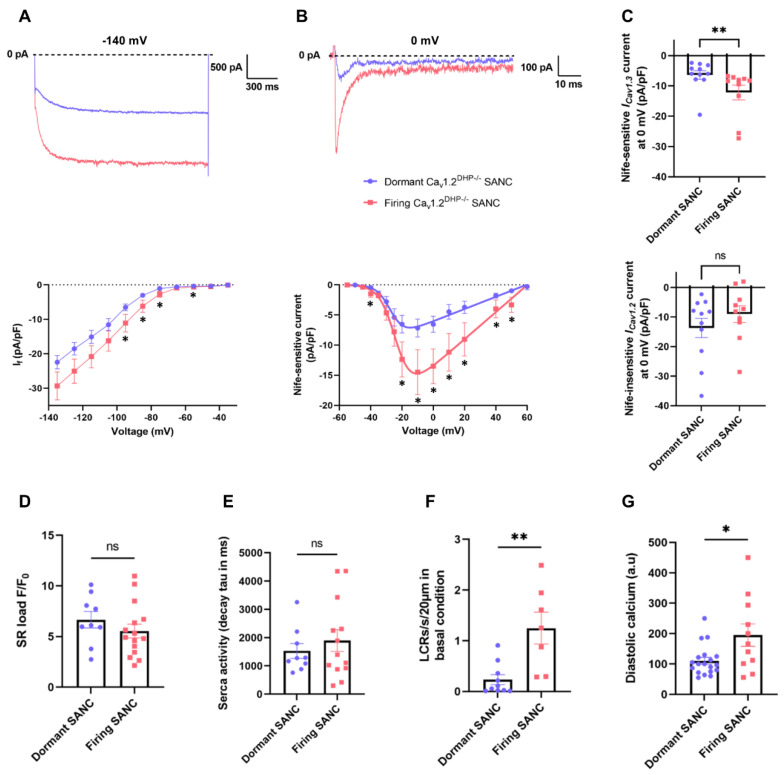
Dormant SANC express reduced *I_Cav1.3_* and *I_f_*. Representative traces and I-V curves of *I_f_* ((**A**), n = 10 dormant and n = 8 firing) and Nife-sensitive current (*I_Cav1.3_*) ((**B**), n = 12 dormant and n = 9 firing) in dormant (blue) and firing (red) SANC. (**C**) Nife-sensitive current (*I_Cav1.3_*) and Nife-insensitive current (*I_Cav1.2_*) densities at 0 mV in dormant and firing *Ca_v_1.2^DHP−/−^* SANC. Averaged SR Ca^2+^ load (**D**), time constant of caffeine-induced Ca^2+^ transient (**E**), number of LCRs (**F**) and diastolic Ca^2+^ (**G**), in dormant (blue, n = 9) and firing (red, n = 15) SANC. ns: non-significant, * *p* < 0.05, ** *p* < 0.01 by unpaired *t*-test.

To obtain insights about dormancy or active automaticity at baseline we measured the density of *I_f_* and the net Nife-sensitive *I_CaL_* current mediated by Ca_v_1.3 channels (*I_Cav1.3_* in isolated *Ca_v_1.2^DHP−/−^* SANC) (Figure 6A,B, Supplementary Figure S6). Current-to-voltage (I-V) relationships showed reduced basal *I_f_* and *I_Cav1.3_* in dormant vs. firing *Ca_v_1.2^DHP−/−^* SANC. Interestingly, while *I_Cav1.3_* density was reduced in dormant *Ca_v_1.2^DHP−/−^* SANC, the net Nife-insensitive *I_CaL_* current mediated by Ca_v_1.2 (*I_Cav1.2_*) was similar in dormant and firing *Ca_v_1.2^DHP−/−^* SANC (Figure 6C).

To assess the functional profile of SR Ca^2+^ release and uptake, we investigated SERCA activity by rapid caffeine application (10 mM). There was no significant difference in SR Ca^2+^ load or in the decay time constant (tau) of the caffeine-induced Ca^2+^ transient between dormant and firing SANC (Figure 6D,E). In contrast, data obtained from previous 2D line-scan experiments (Table 2) showed that the number of LCRs in dormant SANC is decreased by sixfold compared with firing SANC (dormant: 0.23 ± 0.1; firing: 1.25 ± 0.31 LCRs/s/20 µm) and that dormant SANC have significantly lower diastolic intracellular Ca^2+^ (Figure 6F,G). In conclusion, these data suggest that reduced densities of *I_f_* and *I_Cav1.3_*, translating into reduced available inward current during diastolic depolarization and reduced diastolic LCR events at baseline, could contribute to dormancy in SANC.

### 3.7. Expression of Ca_v_1.3 and HCN4 in Intact SAN and Isolated SANC

Since voltage-clamp recordings of *I_f_* and *I_Cav1.3_* showed differential current densities in dormant vs. firing SANC, we wondered if the distribution of Ca_v_1.3 varied in isolated SANC and in the SAN.

**Figure 7:**
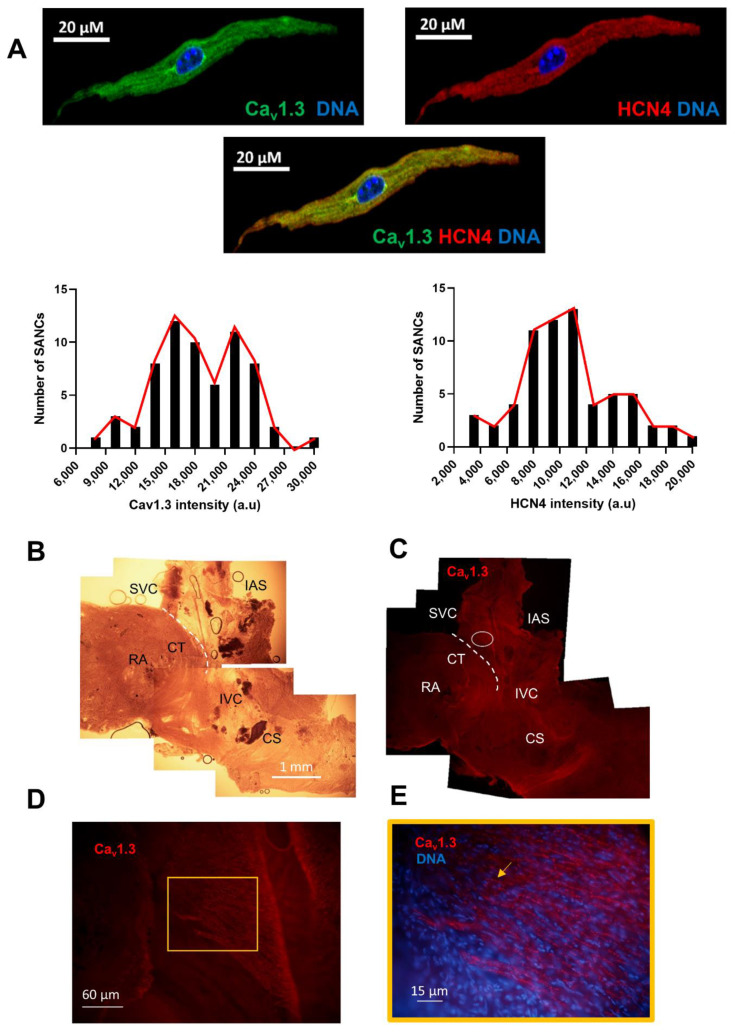
Expression of Ca_v_1.3 and HCN4 in mouse SAN. (**A**) Immunolabeling of Ca_v_1.3 (green) and HCN4 (red) in n = 64 isolated SANC with distribution of SANC based on Ca_v_1.3 or HCN4 fluorescence intensity. Low fluorescence intensity is related to a lower expression of the channels while high density is related to a higher expression. (**B**) Bright-field image of a SAN-RA (N = 3) preparation used for immunolabeling of Ca_v_1.3. (**C**) Immunolabeling of Ca_v_1.3 (red) of the preparation in (**B**). (**D**) Immunolabeling of Ca_v_1.3 in the enlarged region corresponding to the white circle in (**C**). (**E**) Close-up view of Ca_v_1.3-expressing cells (red), together with nuclear labeling of DNA (blue), in the field corresponding to the central square in panel (**D**). The yellow arrow indicates Ca_v_1.3 immunoreactive cells with punctate staining. Abbreviations: RA, right atrium; SVC, superior *vena cava*; IVC, inferior *vena cava*; IAS, inter-atrial septum; CS, coronary sinus (cut-open view); CT, *crista terminalis*. The white dashed line roughly indicates the CT boundary.

**Figure 8:**
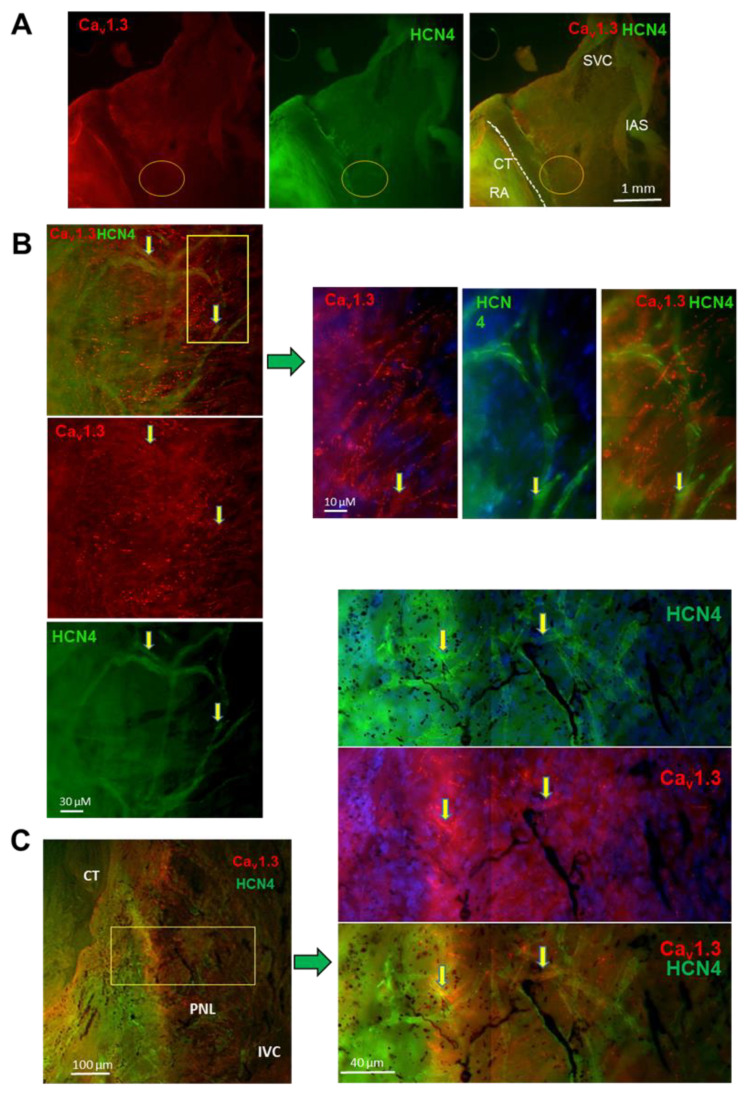
Co-labeling of Ca_v_1.3 and HCN4 in mouse SAN. (**A**) Immunolabeling of Ca_v_1.3 (red) and HCN4 (green) in the isolated cranial SAN-RA preparation. (**B**) Left panels: close-up views of the area circled in (**A**); right panels: enlargement with cells expressing Ca_v_1.3 or HCN4, from boxed region in left panel. (**C**) Immunolabeling of regions co-expressing Ca_v_1.3 and HCN4. The left panel shows the region in accordance with the posterior nodal extension (PNL). The yellow rectangle identifies the region considered for close-up views shown in the left panels. Left panels, from top to bottom, indicate corresponding Ca_v_1.3 and HCN4 immunostaining. Yellow arrows indicate SANC co-expressing Ca_v_1.3 and HCN4.

Co-immunostaining of isolated SANC with anti-HCN4 and anti-Ca_v_1.3 antibodies showed the bimodal distribution of anti-Ca_v_1.3 staining intensity, suggesting the existence of two populations of SANC with a low or high expression of Ca_v_1.3 channels. In contrast, the expression of HCN4 was found centered over a predominant population of isolated SANC (Figure 7A). To compare the expression of Ca_v_1.3 and HCN4 in the intact SAN, we performed immunostaining of SAN tissue with the right atrium. We first mapped the spatial distribution of Ca_v_1.3 immunoreactivity within the SAN (Figure 7B–E, supplementary Figure 7A). Ca_v_1.3 immunoreactivity was widely distributed within the SAN (Figure 7C). Both the cranial (close to the SVC) and the caudal (close to the IVC) SAN regions showed arrays of Ca_v_1.3-expressing cells (Figure 7C,D, Supplementary Figure S7B). Ca_v_1.3 immunoreactivity was present at the cell membrane in aligned “hot spots” of expression (Figure 7D,E). Strong Ca_v_1.3 expression was also observed in the cranial SAN (white circle in Figure 7C), in a sub-region that we have previously shown to be important for the initiation of SAN automaticity [27]. The distribution pattern of Ca_v_1.3 expression in the SAN differed from that of HCN4. Indeed, HCN4 channels were robustly expressed along the *crista terminalis*, a pattern which encompasses the cranial and caudal SAN (Supplementary Figure S7C,D). In addition, arrays of HCN4-positive cells extended from the inter-caval axis toward the direction of the interatrial septum (Supplementary Figure S7C,D), in regions strongly expressing Ca_v_1.3. Comparatively, the arrays of cells in the central region between the crista terminalis and the interatrial septum showed a high expression of Ca_v_1.3 channels (Figure 7C). We thus attempted to study co-expression of Ca_v_1.3 and HCN4 channels in the intact SAN. Co-staining of Ca_v_1.3 and HCN4 channels in the intact SAN-RA was technically challenging and provided images with attenuated fluorescence in comparison to individual staining of the two proteins, possibly because of steric interference between the two antibodies targeting sites in the proximity of each other. Nevertheless, co-staining of the SAN with anti-Ca_v_1.3 and anti-HCN4 antibodies (Figure 8) showed territories co-expressing Ca_v_1.3 and HCN4 in the SAN center in accordance with the expected pacemaker leading site (Figure 8A). This region presented arrays of intermingled cells expressing strong Ca_v_1.3 or HCN4 immunoreactivity (Figure 8B). Cells co-expressing Ca_v_1.3 and HCN4 could be identified in the caudal SAN in accordance with the posterior nodal extension (arrow in Figure 8C).

In summary, the mouse SAN robustly expressed Ca_v_1.3 in areas where the pacemaker leading site was shown to form. In addition, the pattern of expression of Ca_v_1.3 and HCN4 appeared to be different throughout the SAN. These data suggest that while dormant SANC may come from SAN regions expressing low densities of Ca_v_1.3 and HCN4, firing SANC may be situated in regions co-expressing high densities of both Ca_v_1.3 and HCN4.

## 4. Discussion

Our study shows, for the first time, that L-type Ca_v_1.3 channels are critical to maintain the pacemaker activity induced by β-adrenergic activation in dormant SANC. In addition, we show that Ca_v_1.3 channels play a major role in generating a β-adrenergic positive chronotropic effect on pacemaker activity in firing SANC. Finally, we show that the selective pharmacologic inhibition of Ca_v_1.3 channels disrupts the activation of SANC pacemaker activity by hampering the β-adrenergic regulation of LCRs in terms of spatial and time synchronization.

### 4.1. Ca_v_1.3 Channels Are Critical β-Adrenergic Effectors in Maintaining Firing in Dormant SANC

Even though non-pathological SAN exhibits regular firing in vivo, most of the isolated SANC from mice hearts do not show spontaneous activity in vitro (Figure 1 and Figure 2). In this study, we find a similar percentage of isolated firing and non-firing SANC in WT and *Ca_v_1.2^DHP−/−^* mice (Figure 1 and Figure 2). We also find an increased basal slope of the exponential DD phase in firing *Ca_v_1.2^DHP−/−^* SANC, which did not translate into an increased basal firing rate (Supplementary Table S2). The point mutation rendering Ca_v_1.2 channels insensitive to DHPs is in the drug-binding site. Accordingly, no significant differences in gating properties between wild-type and DHP-insensitive Ca_v_1.2 channels have been observed upon heterologous expression in HEK cells [30]. However, we cannot completely exclude that the knock-in of the mutation induces a slight gain-of-function in Ca_v_1.2 channels in the context of the native environment of firing SANC. This would be most likely due to the cellular context, because this difference was not found in dormant SANC that express *Ca_v_1.2^DHP−/−^* channels upon switching to firing mode. Since we recorded similar percentages of dormant SANC in the two genotypes, we conclude that *Ca_v_1.2^DHP−/−^* SANC faithfully recapitulate the pacemaker mechanism of WT cells at baseline.

Previous studies showed that the pharmacological inhibition of *I_Kr_* [34,35], *I_CaL_* [36], PKA [37], CaMKII [38], high concentrations of acetylcholine [39,40] or the combined genetic ablation of Ca_v_1.3 channels with the pharmacologic inhibition of *I_f_* and *I_Na_* [27] could induce dormancy in isolated SANC that were spontaneously firing APs and intracellular Ca^2+^ transients. The mechanism of induction of dormancy may differ, however, because the resting potential of SANC can be set to either positive or negative voltages. For example, while blockade of *I_Kr_* sets the resting potential to positive voltages, the activation of *I_KACh_* arrests the firing of SANC membrane voltages close to the MDP of firing cells [39].

Since dormancy can be induced by a variety of pharmacologic means, it is not surprising to find SANC that are dormant at baseline but can show spontaneous firing when properly stimulated. Kim et al. were indeed the first to report spontaneous firing induced by β-adrenergic activation in isolated dormant SANC from guinea pig hearts [20]. Historically thought of as cells damaged by enzymatic isolation, Kim and co-workers demonstrated that dormant SANC are functional under the stimulation of the cAMP signaling pathway. Here, we show that the automaticity of isolated mouse dormant SANC can be initiated under the activation of β-adrenergic receptors (Figure 1, Figure 2 and Figure 3), showing that the adrenergic stimulation of dormant SANC is shared amongst mammals. We cannot rule out the possibility that some dormant SANC might actually be damaged by enzymatic isolation, especially those that did not respond to β-adrenergic stimulation. However, it has recently been shown that the current densities of *I_f_* and *I_CaL_*, and the repolarizing K^+^ current *I_K_*, are increased under β-adrenergic stimulation in non-responding dormant SANC to the same extent as in responding dormant SANC, showing that cAMP signaling is still functional in this class of SANC [21]. Besides in vitro conditions, the presence of dormant SANC has been observed ex vivo in intact mouse SAN, suggesting that dormant SANC may also be present in vivo [18,19], though their functional physiological role has not yet been fully elucidated.

The transition from dormancy to spontaneous firing is achieved via the enhancement of cAMP-PKA signaling, which causes membrane hyperpolarization to reach the range of voltages spanning diastolic depolarization (−60/−40 mv, Supplementary Figure S2I). Interestingly, the membrane potential of dormant SANC that did not respond to β-adrenergic stimulation was significantly more depolarized than in responding dormant SANC (Supplementary Figure S2H). This suggests that, if a dormant SANC presents with very depolarized resting potential, cAMP-PKA signaling-induced hyperpolarization may not be sufficient to trigger spontaneous firing.

Previous studies demonstrated that *I_Cav1.3_* activates at potentials spanning the diastolic depolarization range (−60 to −40 mV) in mouse SAN cells, while *I_Cav1.2_* activates at voltages from positive to −30 mV [6,25]. Particularly, under ISO, the apparent threshold of *I_Cav1.3_* falls close to −55 mV after stepping from a holding potential of −60 mV [25,26,41]. Second, when mouse SAN action potentials are applied as a voltage-clamp waveform to SAN cells, the genetic ablation of Ca_v_1.3 channels abolished a net *I_Cav1.3_*-mediated current component spanning the whole range of voltages corresponding to diastolic depolarization [29]. This last observation suggests that measuring *I_Cav1.3_* under standard voltage clamp conditions may lead to an underestimation of the contribution of this current to DD. Taken together, these previous works strongly suggest that while *I_Cav1.3_* importantly contributes to the generation of DD, *I_Cav1.2_* is activated during the AP upstroke. This difference in voltage ranges for activation may explain why in both WT and *Ca_v_1.2^DHP−/−^* SANC, nifedipine reverses ISO-mediated AP acceleration in firing SANC, because the contribution to DD of *I_Cav1.3_* would be predominant over that of *I_Cav1.2_* under these conditions.

In line with this previous evidence, we showed that Ca_v_1.3 channels are critical in maintaining β-adrenergic-induced firing in dormant SANC, because the inhibition of Ca_v_1.3 channels arrested spontaneous activity (Figure 2). However, we observed that ISO induced membrane hyperpolarization in dormant SANC showing depolarized membrane potential at rest. In addition, we observed the membrane potential changing to slightly depolarized voltages under Ca_v_1.3 channel inhibition compared with the MDP under β-adrenergic stimulation, yet it did not return to the level of depolarization recorded under the basal condition (Supplementary Figure S2I). These observations are intriguing, because both Ca_v_1.3 and f-(HCN) channels underlie inward currents. Indeed, one would expect ISO to induce membrane depolarization rather than hyperpolarization, by augmenting the activity of Ca_v_1.3 channels and shifting the *I_f_* activation to more positive voltages. While we did not directly investigate the mechanisms underlying the hyperpolarization of dormant SANC upon ISO perfusion, we can speculate that Ca^2+^-activated K^+^ channels [42] may contribute to this process. At a resting potential of −40 mV, the predicted steady-state availability of *I_Cav1.3_* is 20%, which falls at about 10% at −30 mV [6]. A limited but detectable window current is thus expected at resting potentials recorded in depolarized dormant SANC that respond to ISO. In this regard, a PKA-induced increase in *I_Cav1.3_* would lead to enhanced basal Ca^2+^ entry, which in turn could activate Ca^2+^-activated K^+^ channels. Besides *I_Cav1.3_,* Ca_v_1.2-mediated *I_CaL_* may also increase Ca^2+^ entry under ISO to activate Ca^2+^-activated K^+^ channels. However, we cannot exclude that other ion channels contribute to ISO-induced membrane hyperpolarization, such as Ca^2+^-activated Cl^−^ channels. Besides Ca_v_1.3 channels, the cAMP-dependent regulation of f-(HCN) channels could also be involved in switching from dormancy to firing mode, as well as in maintaining automaticity in firing SANCs upon blockade of Ca_v_1.3 channels (Figure 2D). In this regard, we previously reported dormancy in SANC expressing a dominant negative HCN4 subunit which cannot bind cAMP [43]. These dormant cells could be switched to firing mode by perfusion of ISO. Consistent with our results, Fenske et al. [19] recently showed that dormancy could be induced in mouse WT SANC by acute blocking of cAMP binding to HCN4 channels by TAT-TRIP8b_nano_, a peptide that binds to the cyclic nucleotide-binding domain of HCN channels [44].

### 4.2. Ca_v_1.3 Channels Enhance Clock Coupling in Response to β-Adrenergic Stimulation

β-adrenergic stimulation accelerates heart rate by triggering the cAMP-PKA signaling-dependent phosphorylation of actors of both M-clock (Ca_v_1.3, Ca_v_1.2) and Ca^2+^ clock (RyR2, PLB), and the direct regulation of HCN4 channels by cAMP [2,4]. We showed that dormant SANC have reduced *I_f_* and *I_Cav1.3_* densities as well as a reduced number of LCRs [20] and diastolic [Ca^2+^]_i_ compared with firing SANC (Figure 6). It is unlikely that a difference in *I_f_* and *I_Cav1.3_* densities could be solely due to reduced basal cAMP concentration in dormant SANC. Indeed, we find that *I_f_* densities in dormant and firing SANC at hyperpolarized voltages where the current activation variable is close to 1 still tend to be different (Figure 6). This indicates reduced steady-state *I_f_*-mediated membrane conductance that cannot be explained only by differential cAMP levels in the two classes of cells. In the case of *I_Cav1.3_*, the difference in current density between dormant and firing SANC is comparable to the effect of saturating noradrenaline in mouse SANC [25]. Thus, one would have to postulate almost saturating basal levels of cAMP in firing SANC, which is unlikely because ISO strongly increased the firing rate in these cells in a nifedipine-dependent way (Figure 2). In addition, the density of *I_Cav1.2_* is similar in dormant and firing SANC (Figure 6). Finally, we find the differential distribution of both HCN4 and Ca_v_1.3 in isolated SANC and in whole-mount SAN–atria preparations (Figure 7 and Figure 8). In conclusion, previous data from Tsutsui et al. [21] and from our study suggest that the differential expression of both *I_f_* and *I_Cav1.3_* contributes to reduce basal diastolic current in dormant SANC. Interestingly, Ca_v_1.2-mediated *I_CaL_* densities did not differ between dormant and firing SANC, indicating that not all currents are reduced in dormant SANC. The expression of Ca_v_1.2-mediated *I_CaL_* in dormant *Ca_v_1.2^DHP−/−^* SANC indicates that Nife-induced dormancy is not caused by the total blockade of *I_CaL_*. Therefore, our data show that decreased availability in key diastolic currents (*I_f_* and *I_Cav1.3_*) associated with reduced LCRs was preventing the initiation of spontaneous activity in dormant SANC in the absence of adrenergic activation (Figure 6). This is particularly important since reduced *I_Cav1.3_* will also impair spatial and temporal LCRs generation, leading to dormancy as also predicted by the *coupled clock* model of pacemaking [14]. Interestingly, there was no significant difference in SR Ca^2+^ load and the time constant of the caffeine-induced Ca^2+^ transient between dormant and firing SANC, suggesting the normal activity of SERCA. We did not look at pPLB/PLB total ratio between the two cell types, but Tsutsui et al. [21] reported a diminished ratio in dormant SANC in guinea pig. We hypothesize that the same occurs in dormant mouse SANC and that SERCA activity is indeed reduced due to increased PLB inhibition. This difference in phosphorylation levels between dormant and firing SANC could be explained by a decrease in basal cAMP in dormant SANC. In SANC, basal cAMP is tightly regulated by AC and phosphodiesterases (PDE) [45]; hence, it is possible that dormant SANC have higher basal PDE activity.

β-adrenergic stimulation enables the initiation of firing in dormant SANC by increasing basal cAMP and therefore the PKA-dependent phosphorylation of ion channels and of proteins controlling intracellular Ca^2+^ dynamics. The phosphorylation of these proteins is required to maintain firing in dormant SANC because even though dormant SANC can spontaneously fire when an artificial current is injected to reach the DD voltage range, this firing cannot be sustained and immediately stops the second the current is removed (Figure 2). This shows that bringing membrane potential to the firing range (DD voltage range) is not sufficient to sustain automaticity and that PKA-dependent phosphorylation is indispensable. Among the actors phosphorylated by cAMP-PKA signaling, Ca_v_1.3 channels seem to play a central role as regulators of β-adrenergic-induced spontaneous activity. Indeed, in dormant SANC, firing cannot be sustained upon Ca_v_1.3 inhibition. In line with this, in SANC that are spontaneously firing at baseline, blockade of Ca_v_1.3 upon β-adrenergic stimulation decreased the firing rate back to the basal level (Figure 2). Then, both in dormant and firing SANC, Ca_v_1.3 inhibition brings the cells back to their basal state (dormancy in dormant SANC and basal firing rate in firing SANC) as if the cells had “no memory” of being under β-adrenergic stimulation. Thus, even if it can be assumed that other effectors such as PLB are still in their phosphorylated state under continuous Ca_v_1.3 inhibition, this is apparently not sufficient to maintain fast pacemaking.

Our study shows that the activity of Ca_v_1.3 channels constitutes a critical factor for maintaining spontaneous activity in dormant SANC and a high frequency of firing in active SANC under β-adrenergic activation. These data are indicative of an important role for Ca_v_1.3 channels in the β-adrenergic regulation of SANC firing. Nevertheless, other mechanisms are potentially involved in the β-adrenergic regulation of pacemaker activity as a whole under different physiological conditions. Indeed, we reported previously that the systemic non-conditional genetic ablation of Ca_v_1.3 channels in knockout mice (*Ca_v_1.3^−/−^*) does not prevent the ISO-induced acceleration of heart rate in vivo [46] and in firing SANC [29]. On one hand, these observations indicate that other mechanisms that are phosphorylated by cAMP-PKA pathway can compensate for Ca_v_1.3 loss of function. f-channels, the cardiac NCX and RyR-dependent Ca^2+^ release are obvious candidates to mediate the β-adrenergic regulation of SANC firing in the absence of Ca_v_1.3 channels. On the other hand, studies in mice and humans show that the loss of function of the cAMP-dependent regulation of HCN4 channels is also not required for heart rate regulation by the autonomic nervous system [19,43,47]. The conclusion that HCN4 channels are not critical for the chronotropic response in mouse is supported by two other studies on inducible Hcn4 knockout mice [48,49] or by the cardiac-specific and time-dependent expression of a dominant-negative HCN4 subunit carrying silenced conductance [50]. It is thus tempting to speculate that Ca_v_1.3 channels may cooperate with NCX and RyR-dependent Ca^2+^ release to increase pacemaking upon β-adrenergic regulation in firing SANC after the loss of function of f-channels.

### 4.3. Ca_v_1.3 Channels Link M-Clock and Ca^2+^-Clock by Regulating LCRs

We showed in a previous study that the genetic ablation of Ca_v_1.3 Ca^2+^ decreases LCR numbers in SANC from *Ca_v_1.3^−/−^* mice [29]. Here, we provide important pharmacologic evidence with the selective inhibition of *I_Cav1.3_* using *Ca_v_1.2^DHP−/−^* SANC. Indeed, blockade of *I_Cav1.3_* decreased the number and size of LCRs and disrupted spatiotemporal synchronization in late diastole (Figure 4, Table 2). It has been demonstrated by numerical simulations that the synchronization of LCRs and augmented integral LCR Ca^2+^ signal during DD is required along increased sarcolemmal currents (*I_f_*, *I_CaL_*) to accelerate AP firing rate in response to β-adrenergic stimulation [14,51]. Our results are consistent with this model of the increased synchronization of LCRs over time in response to β-adrenergic stimulation. In contrast, upon the inhibition of Ca_v_1.3 channels, LCRs began to move away from the Ca^2+^ transient, their mean distance increasing, whereas the proportion of LCRs close to the transient (<20 ms) decreased, indicating that Ca_v_1.3 channels regulate LCR synchronization (Figure 4). Concomitantly with the abolition of inward *I_Cav1.3_* Ca^2+^ and sustained inward (*I_st_*) [26] current, extreme LCR desynchronization could be an important contributing factor for arrested firing in dormant SANC, as LCRs began to desynchronize right before the cell stopped (Supplementary Figure S4). Important evidence of a direct link between Ca_v_1.3 channels and LCRs is that the desynchronization of LCRs by the inhibition of Ca_v_1.3 channels is not dependent on SERCA activity because Nife did not decrease the pS16-PLB/PLB ratio (Supplementary Figure S5). However, we observed a reduction in pThr17-PLB/PLB ratio in ISO + Nife condition that may be induced by the decrease in basal diastolic calcium following the inhibition of Ca_v_1.3 channels. Furthermore, we previously showed that Ca_v_1.3 channels preferentially co-localize with RyR2, suggesting a spatial proximity [28]. Then, LCR synchronization upon β-adrenergic stimulation could either be due to increased *I_Cav1.3_*-mediated Ca^2+^ entry during diastole leading to an enhanced Ca^2+^-induced Ca^2+^ release mechanism, or to conformational rearrangement in RyR2 and Ca_v_1.3 channels upon phosphorylation, or even both.

### 4.4. Physiological Relevance of Dormant SANC In Vivo

If dormant SANC are indeed present in vivo, as suggested by two different studies [18,19], what could their physiological importance be? Hennis et al. [52], suggested that dormant SANC could act as “brakes” on neighboring firing cells through tonic electrical interactions via gap junction. However, they based their hypothesis on their observation of hyperpolarized (−75 mV) dormant cells when the cAMP-dependent regulation of HCN4 channels is abolished [19]. Here, we observed a majority of dormant SANC, with the preserved cAMP-dependent regulation of HCN4 channels that were depolarized (−35 mV), even though some cells were hyperpolarized (−70 mV) (Supplementary Figure S1). Heterogeneity in the whole SAN tissue in terms of cell behavior, shapes and protein expression has been previously described [2,53,54]. The leading pacemaker site of SAN permanently shifts upon different external stimuli such as adrenergic and cholinergic stimulation, conferring to the SAN adaptability to several constraints [55]. We show here that there is a different spatial distribution of Ca_v_1.3 and HCN4 channels within the entire SAN and even among SANC (Figure 7). This suggests the attractive possibility that different pacemaking mechanisms exist within the SAN. For example, why does Ca_v_1.3 inhibition stop firing in dormant SANC, while it only decreased the frequency back to a basal level in firing SANC? This may be explained by imagining a firing mechanism mostly based on Ca_v_1.3 in dormant SANC, while Ca_v_1.3 channels are important for a positive β-adrenergic chronotropic effect in firing SANC. In line with the idea of different firing mechanisms is that even if the firing rate is the same in dormant and firing SANC under β-adrenergic stimulation, AP characteristics are significantly different between the two cell types (Supplementary Figure S2). This shows that firing in dormant SANC can reach a high frequency and serve as subsidiary pacemaker cells when all SANC are needed to generate a chronotropic response, while having a different pattern of expression in the actors of pacemaking (Ca_v_1.3, HCN4). It is thus possible that not all SANC contribute to heartbeat at the same time, as suggested for the cat SAN [56]. However, SANC with different ion channel expression span the whole SAN and represent a reserve pool of SANC capable of reaching normal frequency upon multiple external stimuli, ensuring SAN adaptability. Indeed, a recent study showed that all SANC with various basal rates tend to reach the same frequency under β-adrenergic stimulation [23]. In this regard, our data indicate that in dormant SANC, Ca_v_1.3 channels are essential to reach this synchronized average rate among SANC during the fight-or-flight response.

## 5. Conclusions

Our results demonstrate that the switch from dormancy to firing mode upon β-adrenergic stimulation in SANC is reliant upon Ca_v_1.3 channels. We show that Ca_v_1.3 channels are an important mediator of the positive chronotropic response to ISO in firing mouse SANC. Our study raises the possibility of different pacemaking mechanisms among SANC, allowing the adaptability of the SAN to different external stimuli while maintaining a physiologically fit beating rate. However, future studies will be needed to address the importance of the phosphorylation of Ca_v_1.3 channels in the generation of basal and β-adrenergic-stimulated pacemaker activity in SANC, and to distinguish it from the effects of selective pharmacologic blockade of the current as shown in our study. Likewise, it will be important to study the importance of the β-adrenergic-activated phosphorylation of RyRs in sustaining firing in SANC. The importance of Ca_v_1.3 channels in the regulation of SANC firing rate could help explain the age-dependent decline in maximal heart rate, especially under exercise. The inhibition of the Ca_v_1.3-dependent β-adrenergic regulation of heart rate may constitute an additional mechanism underlying bradycardia induced by the administration of β-blockers. Conversely, selective inhibitors of Ca_v_1.3 channels may help to reduce heart rate under conditions of inappropriate sinus tachycardia.

## Figures and Tables

**Table 1:** Properties of action potential and diastolic depolarization in *Ca_v_1.2^DHP−/−^* SANC.

	Firing *Ca_v_1.2^DHP−/−^* SANC (n = 21)			Responder Dormant *Ca_v_1.2^DHP−/−^* SANC (n = 12)		
	Baseline	ISO	ISO + Nife	Baseline	ISO	ISO + Nife
Frequency (AP·min^−1^)	197 ± 22	401 ± 15 ****	176 ± 30 ^$$$$^	NA	320 ± 46	49 ± 31
MDP (mV)	−59.8 ± 1.5	−60.3 ± 1.4	−61.7 ± 1.6	NA	−53.4 ± 2	−59.9 ± 3.3
Linear slope (mV·ms^−1^)	0.042 ± 0.005	0.092 ± 0.009 ***	0.053 ± 0.004 ^$$$^	NA	0.080 ± 0.008	0.079 ± 0.005
Expo slope (mV·ms^−1^)	0.317 ± 0.019	0.596 ± 0.042 ****	0.340 ± 0.018 ^$$$$^	NA	0.452 ± 0.046	0.411 ± 0.012
AP threshold (mV)	−43.7 ± 1.5	−42.2 ± 1.5 *	−41.7 ± 1.7	NA	−30.1 ± 2.5	−34.9 ± 2.8
Upstroke (mV·ms^−1^)	13.9 ± 2.8	13.3 ± 2.6	13.4 ± 2.8	NA	6.1 ± 2.9	5.4 ± 0.3
AP amplitude (mV)	82.3 ± 2.9	85.9 ± 3.3	89.1 ± 4	NA	68.3 ± 4	84.1 ± 7.1
AP duration (ms)	133.6 ± 4.4	108.9 ± 3.5 ****	111.4 ± 4.3	NA	117.1 ± 9.1	105.2 ± 15.1
Coefficient of variation	0.42 ± 0.12	0.06 ± 0.01 ****	0.47 ± 0.15 ^$$$^	NA	0.43 ± 0.22	1.11 ± 0.67

Data are expressed as mean ± SEM; MDP, maximum diastolic potential; NA, not applicable. * *p* < 0.05, *** *p* < 0.001, **** *p* < 0.0001 vs. firing SANC at baseline control by one-way ANOVA (parametric) or Friedman test (non-parametric). ^$$$^ *p* < 0.001, ^$$$$^ *p* < 0.0001 vs. firing SANC under ISO perfusion by one-way ANOVA (parametric) or Friedman test (non-parametric). Only n = 3 dormant SANC did not stop under ISO + Nife condition, hence no statistical analysis was performed.

**Table 2:** Properties of LCR in *Ca_v_1.2^DHP−/−^* SANC.

	Firing *Ca_v_1.2^DHP−/−^* SANC (n = 8)			Responder Dormant *Ca_v_1.2^DHP−/−^* SANC (n = 10)		
	Baseline	ISO	ISO + Nife	Baseline	ISO	ISO + Nife
LCR number (LCRs/s/20 µm)	1.25 ± 0.31	1.08 ± 0.18	0.78 ± 0.2	0.23 ± 0.1	1.01 ± 0.24 *	0.41 ± 0.15
LCR size (µm)	4.71 ± 0.39	5.76 ± 0.59	4.28 ± 0.21 ^$^	3.61 ± 0.24	5.98 ± 0.67 *	4.42 ± 0.39
LCR duration (ms)	22.76 ± 3.14	18.83 ± 0.98	19.42 ± 3.01	18.38 ± 2.17	19.35 ± 1.66	17.74 ± 2.26
LCR amplitude (F/F0)	2.32 ± 0.1	2.28 ± 0.12	2.2 ± 0.07	2.43 ± 0.11	2.49 ± 0.12	2.46 ± 0.12

Data are expressed as mean ± SEM; * *p* < 0.05 vs. baseline control by one-way ANOVA (parametric) or Friedman test (non-parametric). ^$^ *p* < 0.05 vs. ISO condition by one-way ANOVA (parametric) or Friedman test (non-parametric).

## Data Availability

All data relevant to this study are included in this article or uploaded as supplementary information.

## Supplementary Materials

The supplementary data are available online at https://www.mdpi.com/article/10.3390/cells11071114/s1. Figure S1. (A) Representative traces of dormant and firing WT SANC under adrenergic stimulation (ISO 100 nM) and subsequent perfusion of Nifedipine (3 µM). (B) Distribution of resting membrane potential of dormant WT SANC. (C) Mean coefficient of variation of WT firing SANC (n = 10) under ISO and ISO + Nife perfusion. (D) Comparison of resting membrane potentials between the WT dormant SANC that initiated firing under adrenergic stimulation (responders, n = 8) and those that did not initiate firing (non-responders, n = 11). * *p* < 0.05 by one-way ANOVA (C) or t-test (D). Figure S2. Comparison of AP parameters under adrenergic stimulation (ISO 100 nM) between dormant (n = 12) and firing (n = 21) Ca_v_1.2^DHP−/−^ SANC (A-G). Data are represented as mean ±SEM. (H) Comparison of membrane voltage between the Ca_v_1.2^DHP−/−^ dormant SANC that initiate firing under adrenergic stimulation (responders) and the ones that did not (non-responders). (I) Comparison of resting membrane potential (Vm) under Tyrode, MDP under ISO 100 nM and Vm under ISO + Nife 3 µM in dormant Ca_v_1.2^DHP−/−^ SANC that stopped firing upon Ca_v_1.3 inhibition. * *p* < 0.05, ** *p* < 0.01, *** *p* < 0.001, **** *p* < 0.0001 by unpaired t-test (A-H) or one-way ANOVA (I). Figure S3. (A) Line-scan and corresponding time-course of the integral of Ca^2+^ fluorescence in dormant Ca_v_1.2^DHP−/−^ SANC that did not respond to β-adrenergic stimulation (n = 3) along with the average number (B), size (C), duration (D) and amplitude (E) of LCRs. Figure S4. (A) Time course of integral of Ca^2+^ fluorescence in an example of dormant Ca_v_1.2^DHP−/−^ SANC that stopped firing under Ca_v_1.3 inhibition. Line-scan and time-course of Ca^2+^ fluorescence of specific time points (a, b and c squares) are represented below. Average distance between LCRs and following Ca^2+^ transient (B) and proportion of LCRs located <20 ms before Ca^2+^ transient in the two Ca_v_1.2^DHP−/−^ SANC in which early effects of Ca_v_1.3 inhibition could be observed. Figure S5. pThr17-PLB/total PLB in isolated SANC from Ca_v_1.2^DHP−/−^ mice incubated with Tyrode (n = 41), ISO 100 nM (n = 31) or ISO + Nife 3 µM (n = 29). Results are from N = 3 mice. * *p* < 0.05 and **** *p* < 0.0001 by one-way ANOVA. Figure S6. Representative traces of *I_f_* (A) and *ICa_v_1.3* (B) currents in dormant (top, blue) and firing (bottom, red) SANC. Voltage steps ranged from −35 mV to −135 mV for *I_f_* (A) and from −55 mV to +60 mV for *ICa_v_1.3* (B). Supplementary Figure S7. (A). Anti Ca_v_1.3 immunostaining of a mouse SAN-RA preparation. (B). Close-up view of anti-Ca_v_1.3 immunofluorescence in a region within the white square in (A). Arrows indicate clusters of Ca_v_1.3 expressing SANC in the SAN region. (C). Sample anti HCN4 immunostaining of the mouse SAN-RA preparation. The image comprises the whole cranial and caudal SAN from the superior (SVC) to inferior (IVC) *venae cavae*. Arrow indicate bundles of HCN4 expressing SAN extending to the central SAN. (D). Close-up view of anti-HCN4 immunofluorescence in a region within the white square in (C). Images are representative of N = 3 SAN-RA preparations stained with anti-Ca_v_1.3 and N = 4 preparations stained with anti-HCN4 antibody. Other abbreviations: CT, *Crista terminalis*; IAS, interatrial septum; PNE, posterior nodal extension. Table S1. Properties of action potential and diastolic depolarization in WT SANC; Table S2. Comparison of action potential and diastolic depolarization properties in WT and Ca_v_1.2^DHP−/−^ SANC.
