# Supplementary material for: L-Type Cav1.3 Calcium Channels Are Required for Beta-Adrenergic Triggered Automaticity in Dormant Mouse Sinoatrial Pacemaker Cells
Source: Cells. 2022 Mar 25;11(7):1114. doi: 10.3390/cells11071114 (PMC8997967; doi:10.3390/cells11071114)
Supplement: Supplementary file 1 [file cells-11-01114-s001.zip › Supplemental material cells v4.pdf]

Supplementary Figures

Supplementary Figure S1. (A) Representative traces of dormant and firing WT SANC under adrenergic stimulation (ISO 100 nM) and subsequent perfusion of Nifedipine (3 μM). (B) Distribution of resting membrane potential of dormant WT SANC. (C) Mean coefficient of variation of WT firing SANC (n=10) under ISO and ISO + Nife perfusion. (D) Comparison of resting membrane potentials between the WT dormant SANC that initiated firing under adrenergic stimulation (responders, n=8) and those that did not initiate firing (non-responders, n=11). *p < 0.05 by one-way ANOVA (C) or t-test (D).

Supplementary Figure S2. Comparison of AP parameters under adrenergic stimulation (ISO 100 nM) between dormant (n=12) and firing (n=21) $Ca_v1.2^{DHP-/-}$ SANC (A-G). Data are represented as mean ±SEM. (H) Comparison of membrane voltage between the $Ca_v1.2^{DHP-/-}$ dormant SANC that initiate firing under adrenergic stimulation (responders) and the ones that did not (non-responders). (I) Comparison of resting membrane potential (Vm) under Tyrode, MDP under ISO 100 nM and Vm under ISO + Nife 3 μM in dormant $Ca_v1.2^{DHP-/-}$ SANC that stopped firing upon $Ca_v1.3$ inhibition. *p < 0.05, **p < 0.01, ***p < 0.001, ****p < 0.0001 by unpaired t-test (A-H) or one-way ANOVA (I).

Supplementary Figure S3. (A) Line-scan and corresponding time-course of the integral of Ca^{2+} fluorescence in dormant $Ca_v1.2^{DHP-/-}$ SANC that did not respond to β-adrenergic stimulation (n=3) along with the average number (B), size (C), duration (D) and amplitude (E) of LCRs.

Supplementary Figure S4. (A) Time course of integral of Ca^{2+} fluorescence in an example of dormant $Ca_v1.2^{DHP-/-}$ SANC that stopped firing under $Ca_v1.3$ inhibition. Line-scan and time-course of Ca^{2+} fluorescence of specific time points (a, b and c squares) are represented below. Average distance between LCRs and following Ca^{2+} transient (B) and proportion of LCRs located < 20 ms before Ca^{2+} transient in the two $Ca_v1.2^{DHP-/-}$ SANC in which early effects of $Ca_v1.3$ inhibition could be observed.

Supplementary Figure S5. pThr17-PLB/total PLB in isolated SANC from $Ca_v1.2^{DHP-/-}$ mice incubated with Tyrode (n=41), ISO 100 nM (n=31) or ISO + Nife 3 μM (n=29). Results are from N=3 mice. *p < 0.05 and ****p < 0.0001 by one-way ANOVA

Supplementary Figure S6. Representative traces of I_f (A) and $I_{Cav1.3}$ (B) currents in dormant (top, blue) and firing (bottom, red) SANC. Voltage steps ranged from -35mV to -135mV for I_f (A) and from -55mV to +60mV for $I_{Cav1.3}$ (B).

Supplementary Figure S7. (A). Anti $Ca_v1.3$ immunostaining of a mouse SAN-RA preparation. (B). Close-up view of anti-$Ca_v1.3$ immunofluorescence in a region within the white square in (A). Arrows indicate clusters of $Ca_v1.3$ expressing SANC in the SAN region. (C). Sample anti HCN4 immunostaining of the mouse SAN-RA preparation. The image comprises the whole cranial and caudal SAN from the superior (SVC) to inferior (IVC) *venae cavae*. Arrow indicate bundles of HCN4 expressing SAN extending to the central SAN. (D). Close-up view of anti-HCN4 immunofluorescence in a region within the white square in (C). Images are representative of N=3 SAN-RA preparations stained with anti-$Ca_v1.3$ and N=4 preparations stained with anti-HCN4 antibody. Other abbreviations: CT, *Crista terminalis*; IAS, interatrial septum; PNE, posterior nodal extension.
