# Supplementary material for: L-Type Cav1.3 Calcium Channels Are Required for Beta-Adrenergic Triggered Automaticity in Dormant Mouse Sinoatrial Pacemaker Cells
Source: Cells. 2022 Mar 25;11(7):1114. doi: 10.3390/cells11071114 (PMC8997967; doi:10.3390/cells11071114)
Supplement: Supplementary file 1 [file cells-11-01114-s001.zip › Supplementary figures v4.pptx]

## Slide 1
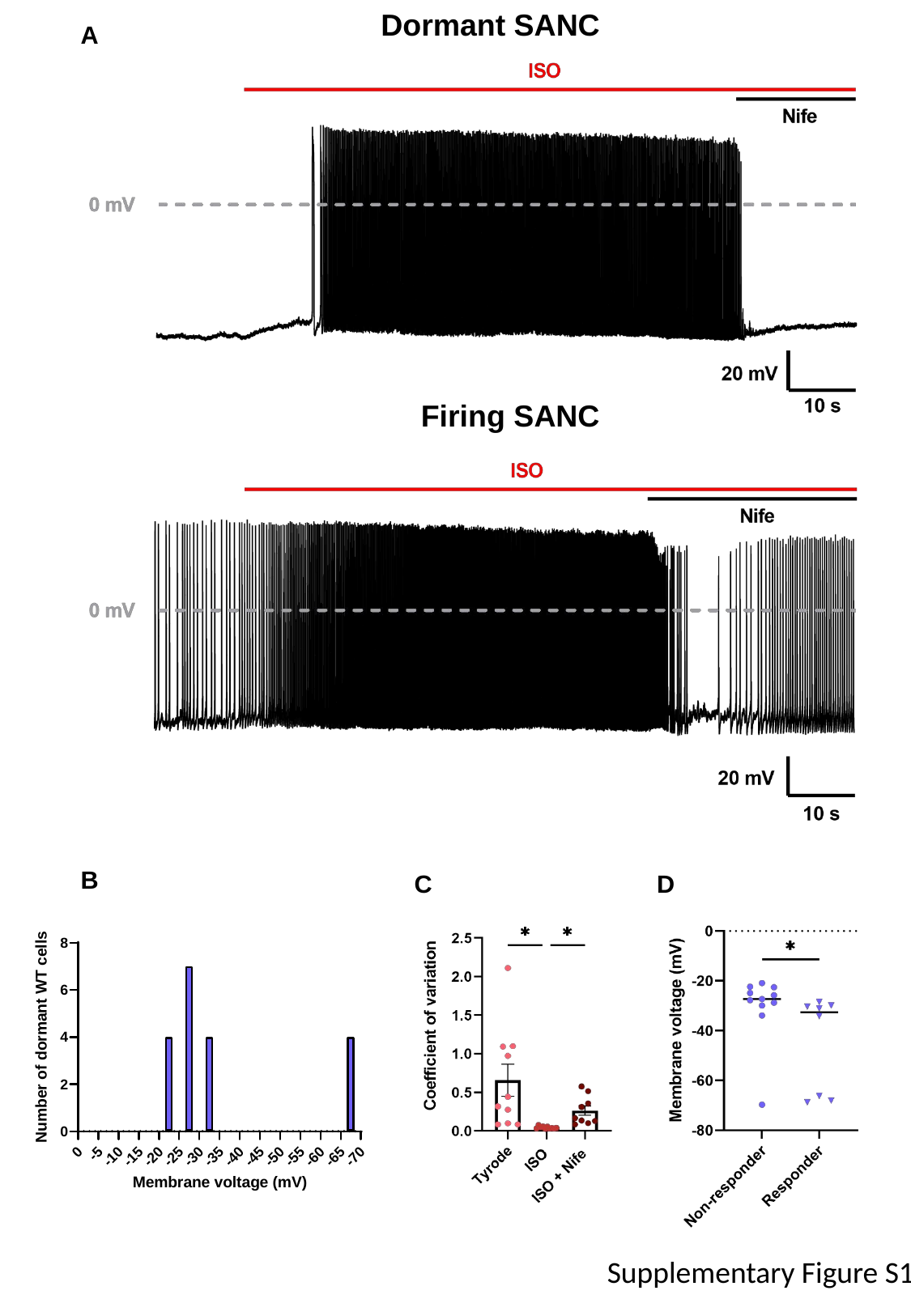

Dormant SANC
A
Firing SANC
B
C
D
Supplementary Figure S1

## Slide 2
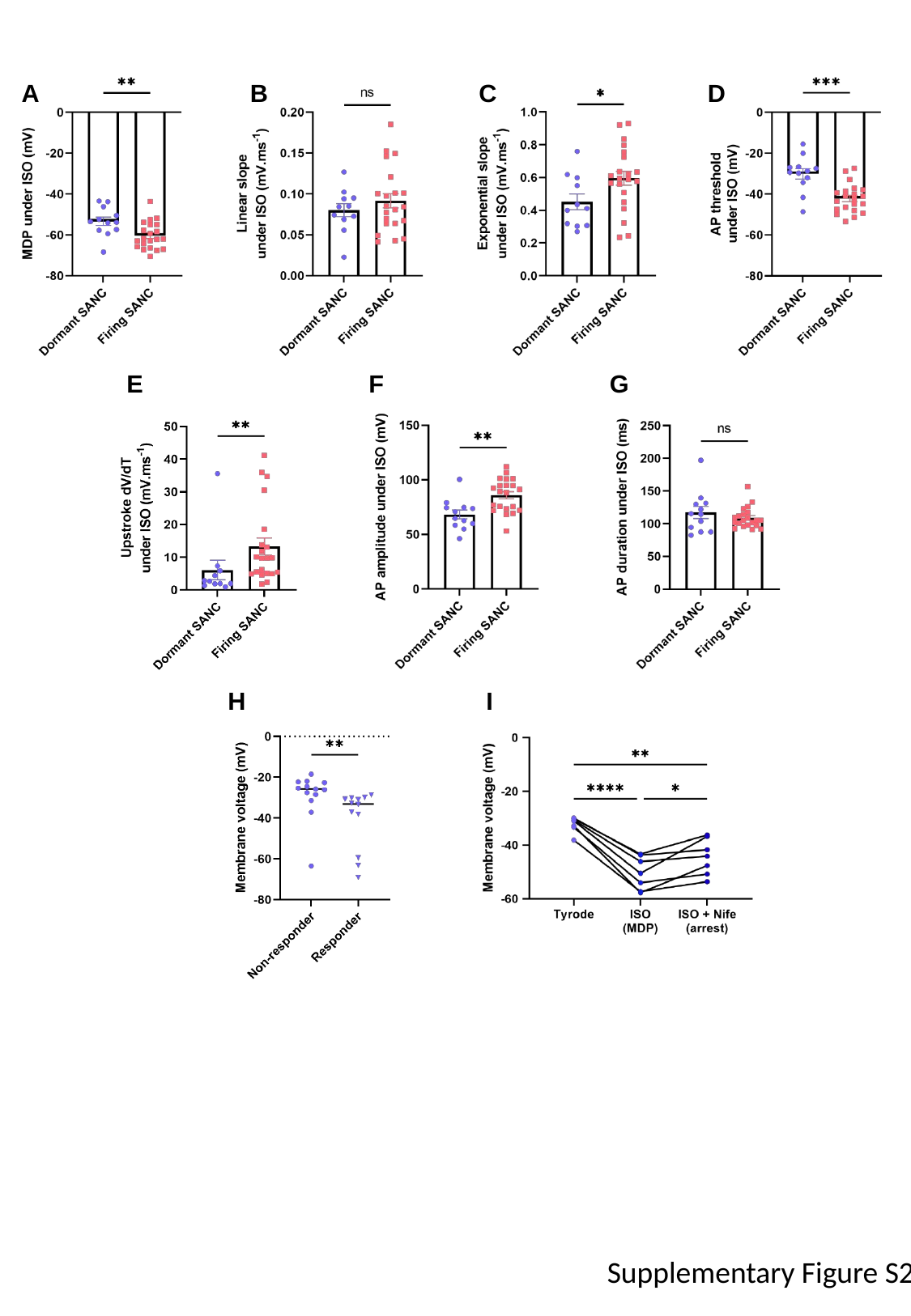

A
B
C
D
E
F
G
H
I
Supplementary Figure S2

## Slide 3
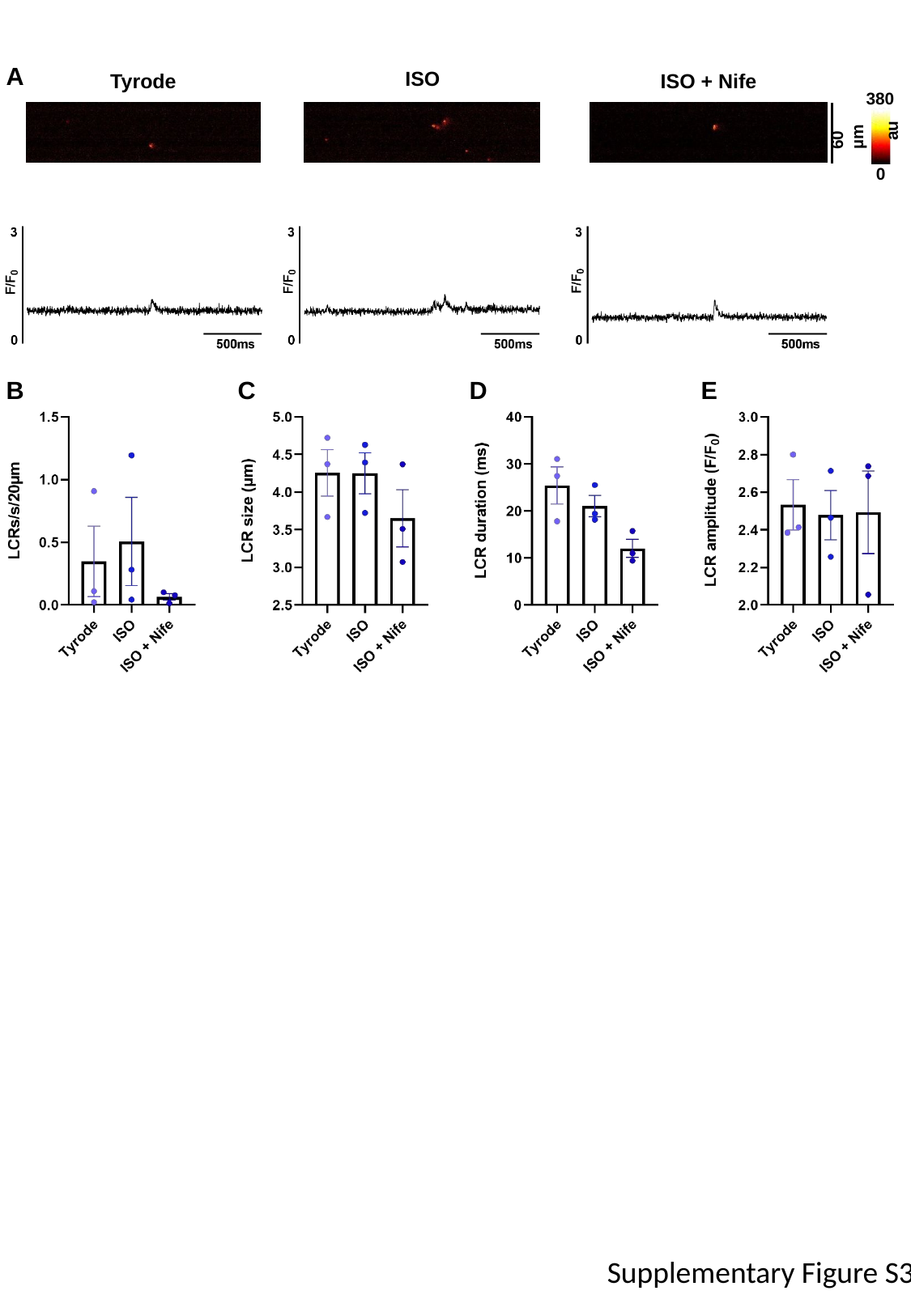

A
ISO
Tyrode
ISO + Nife
380
60 µm
au
0
B
C
D
E
Supplementary Figure S3

## Slide 4
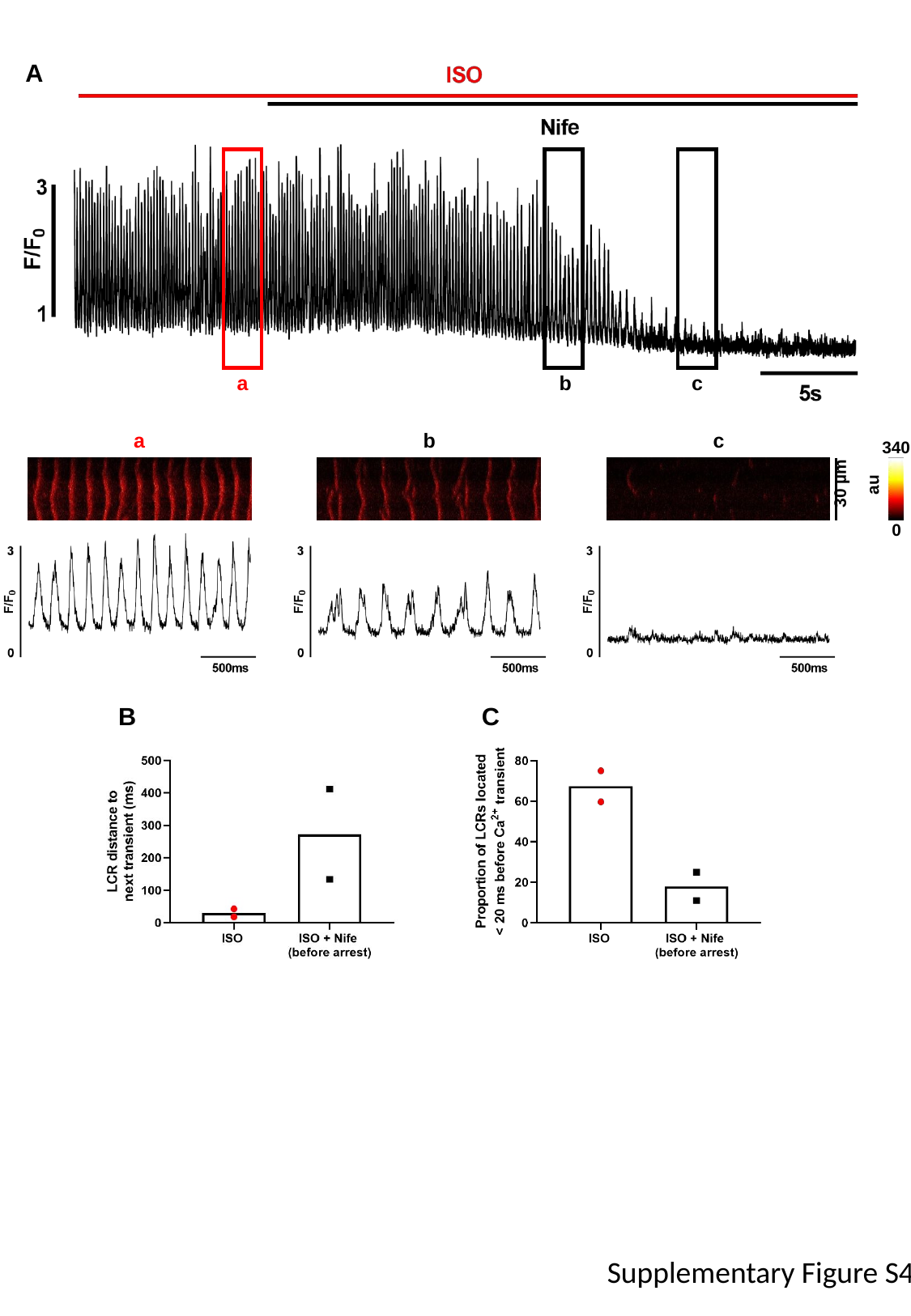

A
a
b
c
a
b
c
340
30 µm
au
0
B
C
Supplementary Figure S4

## Slide 5
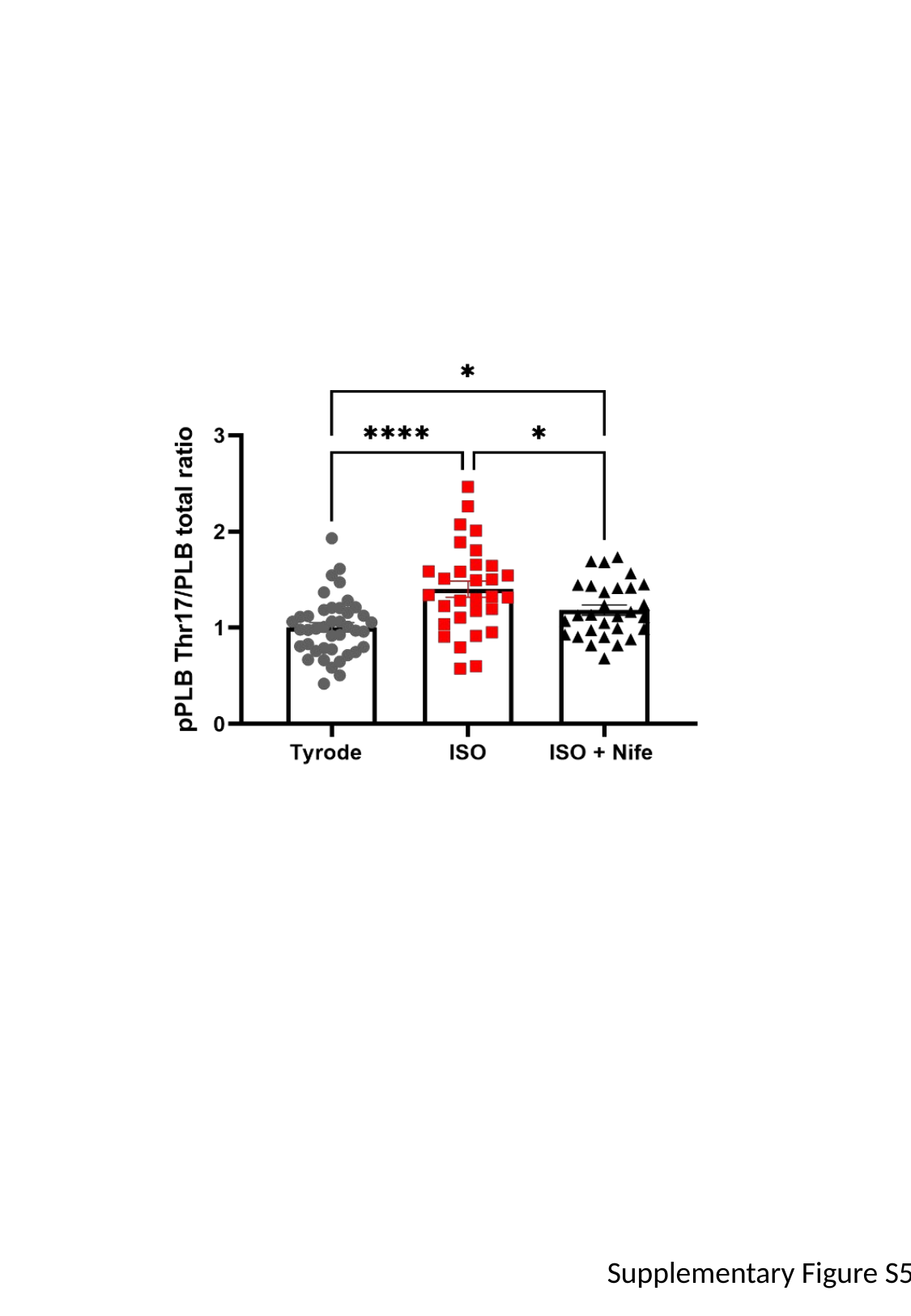

Supplementary Figure S5

## Slide 6
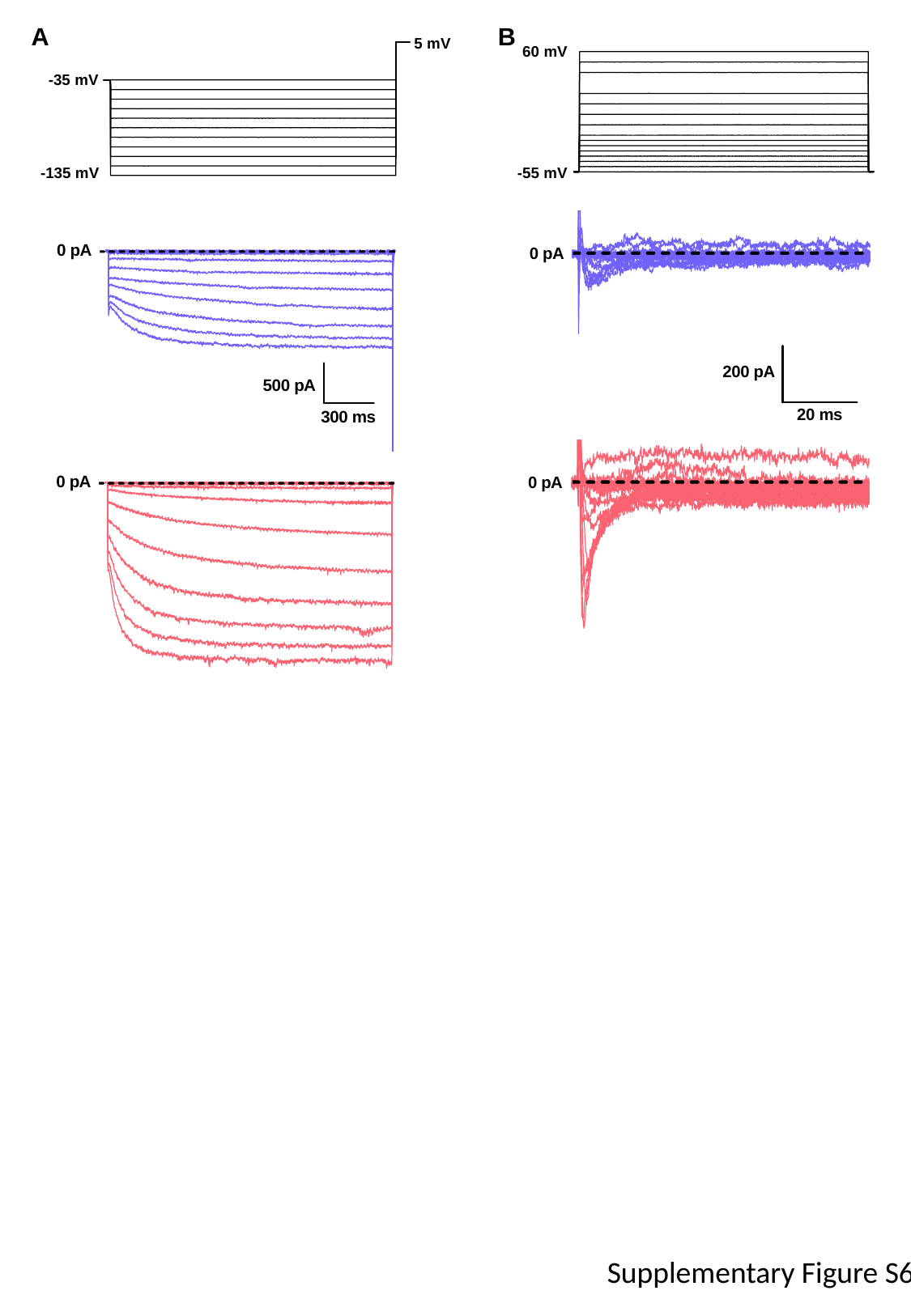

A
B
5 mV
60 mV
-35 mV
-55 mV
-135 mV
Supplementary Figure S6

## Slide 7
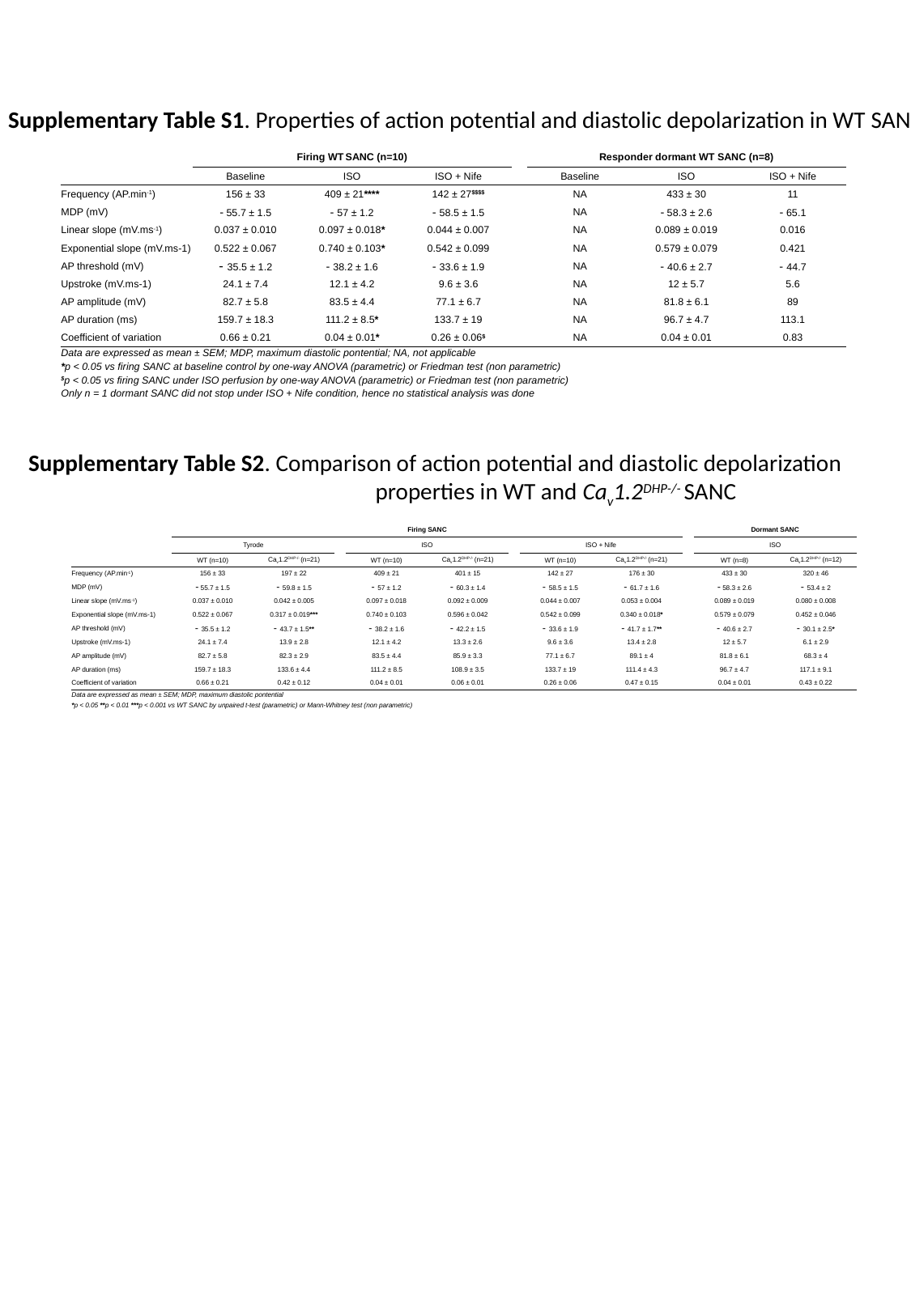

Supplementary Table S1. Properties of action potential and diastolic depolarization in WT SANC
| | Firing WT SANC (n=10) | | | | Responder dormant WT SANC (n=8) | | |
| --- | --- | --- | --- | --- | --- | --- | --- |
| | Baseline | ISO | ISO + Nife | | Baseline | ISO | ISO + Nife |
| Frequency (AP.min-1) | 156 ± 33 | 409 ± 21\*\*\*\* | 142 ± 27$$$$ | | NA | 433 ± 30 | 11 |
| MDP (mV) | - 55.7 ± 1.5 | - 57 ± 1.2 | - 58.5 ± 1.5 | | NA | - 58.3 ± 2.6 | - 65.1 |
| Linear slope (mV.ms-1) | 0.037 ± 0.010 | 0.097 ± 0.018\* | 0.044 ± 0.007 | | NA | 0.089 ± 0.019 | 0.016 |
| Exponential slope (mV.ms-1) | 0.522 ± 0.067 | 0.740 ± 0.103\* | 0.542 ± 0.099 | | NA | 0.579 ± 0.079 | 0.421 |
| AP threshold (mV) | - 35.5 ± 1.2 | - 38.2 ± 1.6 | - 33.6 ± 1.9 | | NA | - 40.6 ± 2.7 | - 44.7 |
| Upstroke (mV.ms-1) | 24.1 ± 7.4 | 12.1 ± 4.2 | 9.6 ± 3.6 | | NA | 12 ± 5.7 | 5.6 |
| AP amplitude (mV) | 82.7 ± 5.8 | 83.5 ± 4.4 | 77.1 ± 6.7 | | NA | 81.8 ± 6.1 | 89 |
| AP duration (ms) | 159.7 ± 18.3 | 111.2 ± 8.5\* | 133.7 ± 19 | | NA | 96.7 ± 4.7 | 113.1 |
| Coefficient of variation | 0.66 ± 0.21 | 0.04 ± 0.01\* | 0.26 ± 0.06$ | | NA | 0.04 ± 0.01 | 0.83 |
| Data are expressed as mean ± SEM; MDP, maximum diastolic pontential; NA, not applicable | | | | | | | |
| \*p < 0.05 vs firing SANC at baseline control by one-way ANOVA (parametric) or Friedman test (non parametric) | | | | | | | |
| $p < 0.05 vs firing SANC under ISO perfusion by one-way ANOVA (parametric) or Friedman test (non parametric) | | | | | | | |
| Only n = 1 dormant SANC did not stop under ISO + Nife condition, hence no statistical analysis was done | | | | | | | |
Supplementary Table S2. Comparison of action potential and diastolic depolarization
 properties in WT and Cav1.2DHP-/- SANC
| | Firing SANC | | | | | | | | | Dormant SANC | |
| --- | --- | --- | --- | --- | --- | --- | --- | --- | --- | --- | --- |
| | Tyrode | | | ISO | | | ISO + Nife | | | ISO | |
| | WT (n=10) | Cav1.2DHP-/- (n=21) | | WT (n=10) | Cav1.2DHP-/- (n=21) | | WT (n=10) | Cav1.2DHP-/- (n=21) | | WT (n=8) | Cav1.2DHP-/- (n=12) |
| Frequency (AP.min-1) | 156 ± 33 | 197 ± 22 | | 409 ± 21 | 401 ± 15 | | 142 ± 27 | 176 ± 30 | | 433 ± 30 | 320 ± 46 |
| MDP (mV) | - 55.7 ± 1.5 | - 59.8 ± 1.5 | | - 57 ± 1.2 | - 60.3 ± 1.4 | | - 58.5 ± 1.5 | - 61.7 ± 1.6 | | - 58.3 ± 2.6 | - 53.4 ± 2 |
| Linear slope (mV.ms-1) | 0.037 ± 0.010 | 0.042 ± 0.005 | | 0.097 ± 0.018 | 0.092 ± 0.009 | | 0.044 ± 0.007 | 0.053 ± 0.004 | | 0.089 ± 0.019 | 0.080 ± 0.008 |
| Exponential slope (mV.ms-1) | 0.522 ± 0.067 | 0.317 ± 0.019\*\*\* | | 0.740 ± 0.103 | 0.596 ± 0.042 | | 0.542 ± 0.099 | 0.340 ± 0.018\* | | 0.579 ± 0.079 | 0.452 ± 0.046 |
| AP threshold (mV) | - 35.5 ± 1.2 | - 43.7 ± 1.5\*\* | | - 38.2 ± 1.6 | - 42.2 ± 1.5 | | - 33.6 ± 1.9 | - 41.7 ± 1.7\*\* | | - 40.6 ± 2.7 | - 30.1 ± 2.5\* |
| Upstroke (mV.ms-1) | 24.1 ± 7.4 | 13.9 ± 2.8 | | 12.1 ± 4.2 | 13.3 ± 2.6 | | 9.6 ± 3.6 | 13.4 ± 2.8 | | 12 ± 5.7 | 6.1 ± 2.9 |
| AP amplitude (mV) | 82.7 ± 5.8 | 82.3 ± 2.9 | | 83.5 ± 4.4 | 85.9 ± 3.3 | | 77.1 ± 6.7 | 89.1 ± 4 | | 81.8 ± 6.1 | 68.3 ± 4 |
| AP duration (ms) | 159.7 ± 18.3 | 133.6 ± 4.4 | | 111.2 ± 8.5 | 108.9 ± 3.5 | | 133.7 ± 19 | 111.4 ± 4.3 | | 96.7 ± 4.7 | 117.1 ± 9.1 |
| Coefficient of variation | 0.66 ± 0.21 | 0.42 ± 0.12 | | 0.04 ± 0.01 | 0.06 ± 0.01 | | 0.26 ± 0.06 | 0.47 ± 0.15 | | 0.04 ± 0.01 | 0.43 ± 0.22 |
| Data are expressed as mean ± SEM; MDP, maximum diastolic pontential | | | | | | | | | | | |
| \*p < 0.05 \*\*p < 0.01 \*\*\*p < 0.001 vs WT SANC by unpaired t-test (parametric) or Mann-Whitney test (non parametric) | | | | | | | | | | | |

## Slide 8
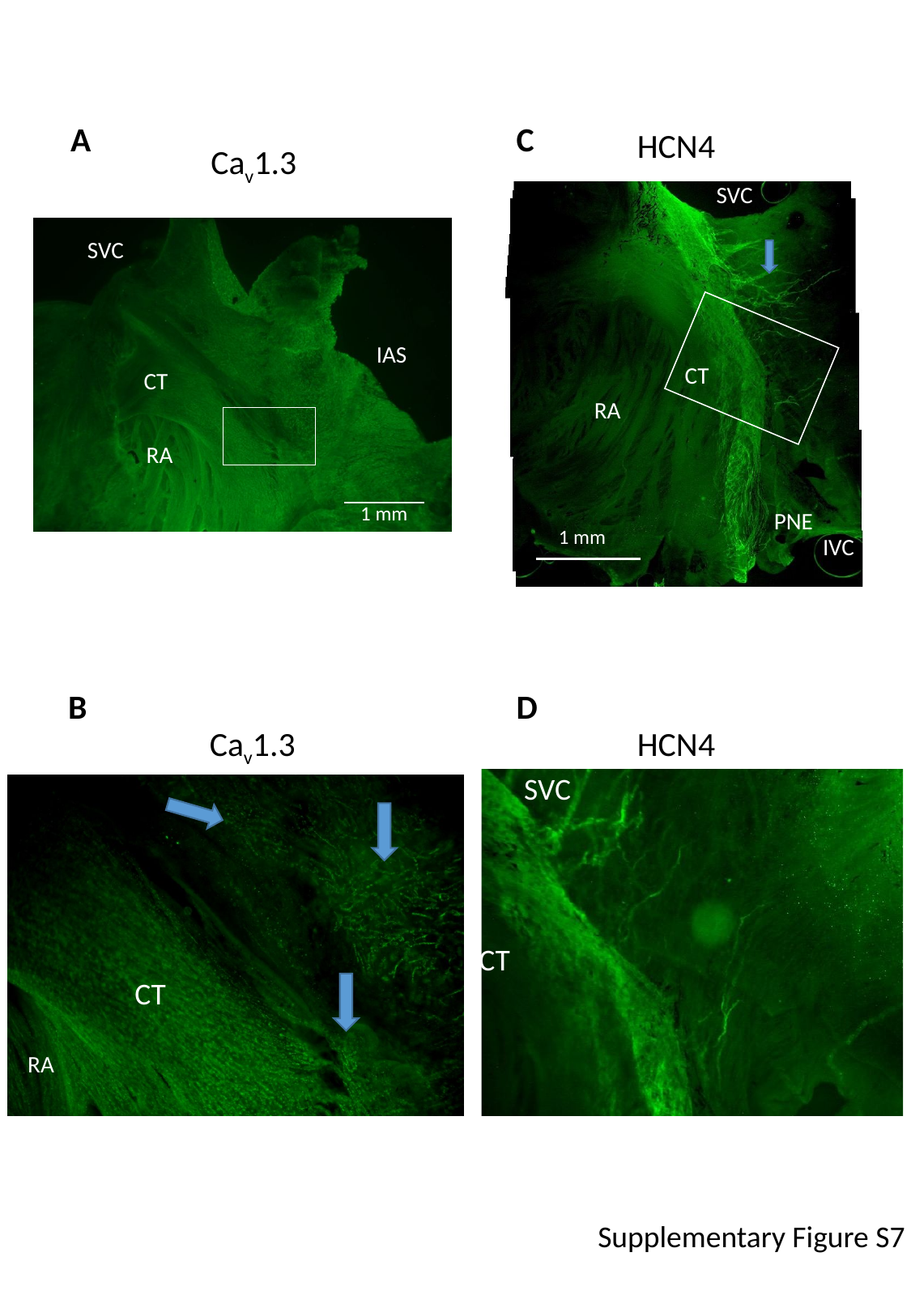

A
C
HCN4
Cav1.3
SVC
CT
RA
IVC
SVC
IAS
CT
RA
1 mm
PNE
1 mm
B
D
Cav1.3
HCN4
SVC
CT
CT
RA
Supplementary Figure S7
